# Highly Sensitive Detection of Tyrosine and Neurotransmitters by Stereoselective Biosynthesis and Photochemically Induced Dynamic Nuclear Polarization

**DOI:** 10.1021/jacs.5c11334

**Published:** 2025-11-05

**Authors:** Ummay Mahfuza Shapla, Jamorious L. Smith, Anubhab Halder, Lillian Thompson, Andrew R. Buller, Silvia Cavagnero

**Affiliations:** † Department of Chemistry, 5228 University of Wisconsin-Madison, 1101 University Ave, Madison, Wisconsin 53706, United States; ‡ Biophysics Program, University of Wisconsin-Madison, Madison, Wisconsin 53706, United States

## Abstract

Tyrosine (Tyr) is a building block of proteins and a precursor of key neurotransmitters including dopamine and epinephrine. Investigations on the metabolic fate of Tyr are hampered by poor sensitivity and resolution, hindering the diagnosis of debilitating diseases including phenylketonuria, tyrosine-hydroxylase deficiency and progressive infantile encephalopathy. Here, we show that Tyr constructs bearing either a quasi-isolated ^1^H^α^–^13^C^α^ spin pair (QISP Tyr) or natural-abundance nuclides are detected at high sensitivity and resolution in biologically relevant media by optically enhanced NMR. QISP Tyr, generated via a chemoenzymatic strategy starting from achiral materials, was quantified at 200 nM and 10 μM levels in aqueous buffer and cell extracts, respectively, via low-concentration photochemically induced dynamic nuclear polarization (LC-photo-CIDNP). Further, natural-abundance epinephrine was revealed at unprecedented 10 nM levels (1.3 nanograms), while Tyr and L-DOPA required 500 nM concentrations. In all, this study establishes the ultrasensitive atomic-resolution detection of Tyr and Tyr-related neurotransmitters by optically enhanced NMR.

## Graphical abstract

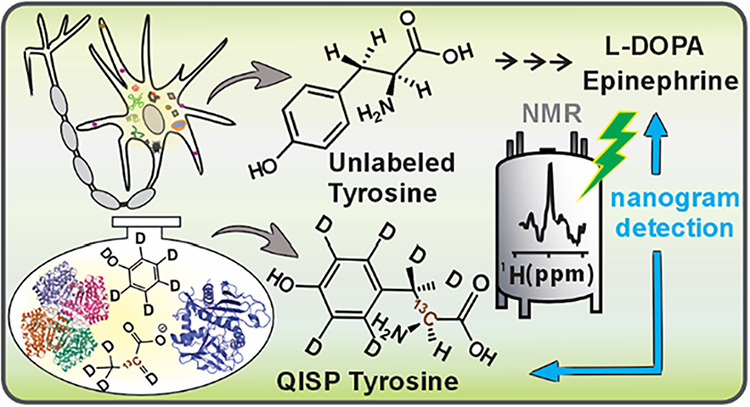

## Introduction

Tyrosine (Tyr) is an aromatic amino acid often found in polypeptides and proteins. As a free species, Tyr is also a crucial metabolite and a precursor to a variety of growth regulators and neurotransmitters in plants and mammals.^1,2^ Efficient analysis of free Tyr and its metabolic fate, including generation of L-DOPA and catecholamines (e.g., epinephrine and dopamine),^3,4^ requires a high-sensitivity and high-resolution approach, as well as the availability of specifically labeled isotopologs. This need is particularly stringent in the context of the medical diagnosis of Tyr-dysfunction diseases including phenylalanine hydroxylase deficiency (a.k.a. phenylketonuria), progressive infantile encephalopathy and tyrosine hydroxylase deficiency (THD), where both Tyr and its metabolic products need to be followed.^5–12^ Further, Tyr-metabolism and biomedical investigations are most efficiently carried out if the desired isotopologs have substitution patterns that can be easily generated depending on specific needs.^13–18^

To date, a detection technique that is at the same time highly sensitive and capable of promptly elucidating atomic-resolution structural details on Tyr and its metabolites *in situ* is not available. Notably, while the currently employed mass spectrometry, HPLC, and biochemical assays are highly sensitive, postsampling manipulations are required (e.g., gas-phase ionization, treatment with organic solvents, addition of binding agents, microextraction),^19^ and direct atomic-resolution information on three-dimensional structure within the natural milieu is missing. This drawback hampers investigations targeting Tyr metabolism in the context of human health and disease, especially in cases where *in situ* assessments of both abundance and conformation are required.

The above challenges can be addressed by synergistically combining a novel modular bioenzymatic route to Tyr isotopologs with an optically enhanced NMR spectroscopy technology known as low-concentration photochemically induced dynamic nuclear polarization (LC-photo-CIDNP).^20–23^ LC-photo-CIDNP is unique in its ability to detect much smaller sample concentrations than conventional NMR, including its photo-CIDNP flavor.^24^ This technology is particularly powerful when LED irradiation sources are employed^24,25^ due to their simplicity and portability. Previous LC-photo-CIDNP studies showed detectability of Trp, either in isolation or within proteins, down to low-μM to sub-μM concentrations.^23,26^ In essence, LC-photo-CIDNP significantly augments the capabilities of the parent photo-CIDNP technique, which has been typically employed to appraise biomolecular solvent-exposure and folding^22,27–30^ at much higher concentration (≥mM). In other words, LC-photo-CIDNP is an emerging technology that offers remarkable opportunities for ultrasensitive and high-resolution structural^21,23,24,26,31–33^ and screening^34,35^ explorations in biology. A particularly useful development was the enhancement of LC-photo-CIDNP sensitivity displayed by a Trp-isotopolog (QISP Trp) bearing a quasi-isolated spin pair (QISP, Figure 1A).^23^

**Figure 1:**
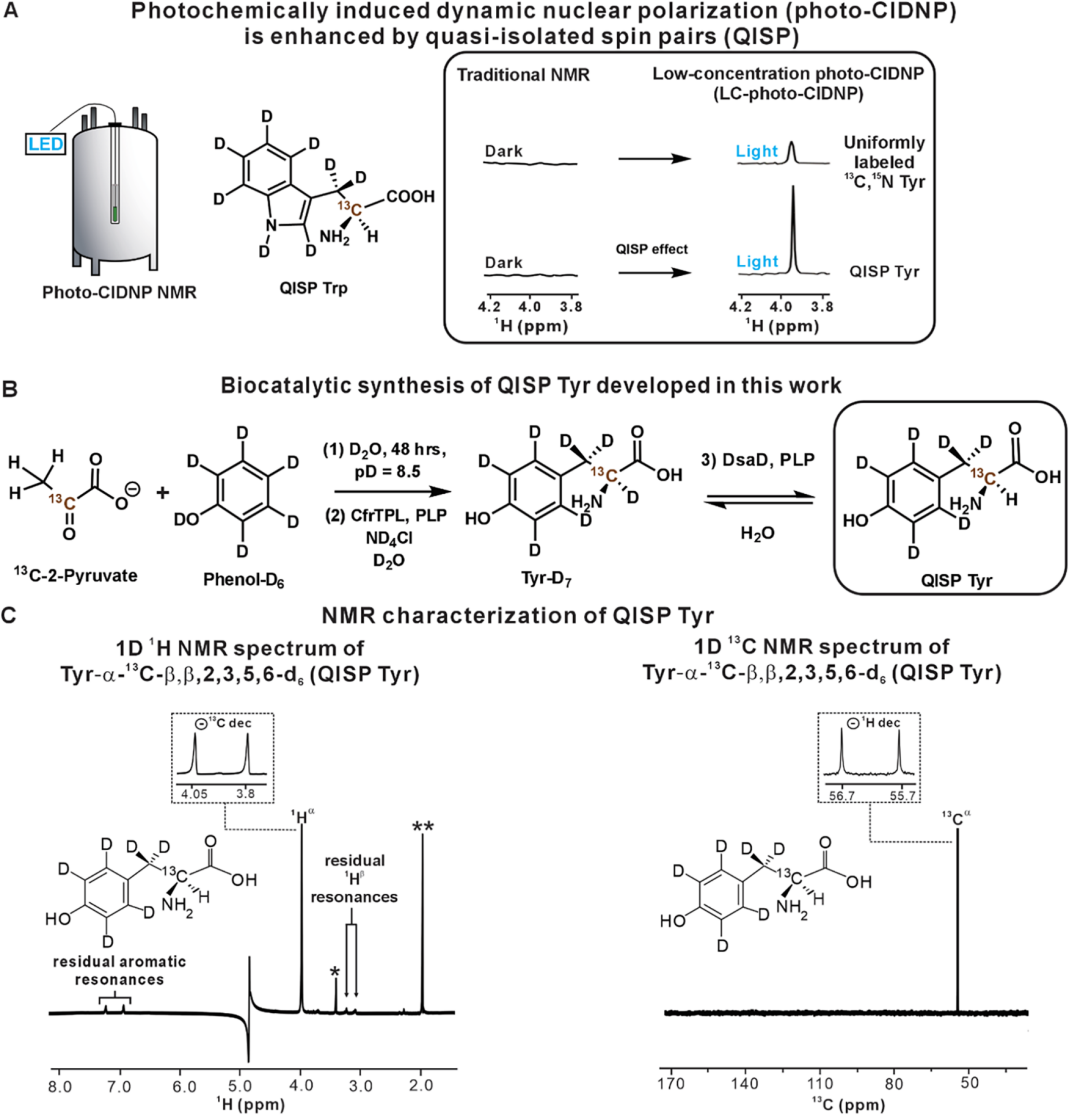
Overview of known LC-photo-CIDNP hyperpolarization advantages and outline of the novel biocatalytic approach to QISP Tyr developed in this work. (A) Comparison between spectral features of traditional NMR and LC-photo-CIDNP in the presence of a quasi-isolated spin pair (QISP). (B) Novel synthetic scheme for the chemoenzymatic preparation of QISP Tyr. The initial reactions (steps 1–2) generate perdeuterated Tyr. The deuteron at C^α^-D is subsequently removed (step 3) via selective H/D exchange. (C) NMR characterization of QISP Tyr. Left: 1D ^1^H NMR spectrum (pulse-acquire with solvent suppression) of QISP Tyr (600 μM, 90/10% H_2_O/D_2_O) in the absence and presence of ^13^C decoupling (*n* = 2). Data collection included 128 scans, and a 5 s recycle delay. The inset shows the ^1^H^α^ region of the spectrum in the absence of ^13^C decoupling. The resonances denoted as * and ** are due to residual methanol and acetate, respectively. Right: 1D ^13^C NMR spectrum (pulse-acquire) of QISP Tyr in the absence and presence of ^1^H decoupling during acquisition (256 scans, 2 s recycle delay). The inset shows the ^13^C^α^ region of the spectrum in the absence of ^1^H decoupling (*n* = 2). All data were collected on a 14.1 T (600 MHz) NMR spectrometer.

In this work, we develop a modular biosynthetic route to a variety of Tyr isotopologs including QISP Tyr, and show that the latter can be readily detected in aqueous solution down to 200 nM levels via LC-photo-CIDNP. Further, we demonstrate that dilute (10 μM) QISP Tyr can be detected in complex biological media (cell extracts). We also establish that the LC-photo-CIDNP approach is capable of efficiently revealing aromatic molecules at natural abundance including Tyr and its catabolites epinephrine and L-DOPA. While unlabeled Tyr and L-DOPA detectability demanded ≥500 nM levels, epinephrine was readily identified even at 10 nM. This is the lowest ever achieved NMR-detectable concentration in solution, to date. Notably, epinephrine analysis in the low-nanomolar range was readily achieved at 14.1 T, (600 MHz), a commonly employed field in biomolecular NMR. In all, the results shown here pave the way to the *in situ* high-resolution and hypersensitive analysis of Tyr and its metabolites in basic science and disease.

## Results

### Preparation of Tyr Isotopologs Including QISP Tyr

The preparation of amino acid isotopologs faces unique constraints relative to standard synthetic approaches, given the need to place desired isotopes at specific positions. We previously reported the preparation of QISP tryptophan (QISP Trp) based on an enzyme cascade reaction.^36^ On the other hand, the synthesis of QISP tyrosine (QISP Tyr) cannot be carried out with similar procedures, and it requires a wholly distinct chemoenzymatic approach. Previous biocatalytic routes to synthesize Tyr isotopologs, including those involving phenylalanine ammonia lyases (PALs),^37,38^ rely on the catalytic reversible elimination of ammonia from l-phenylalanine (Phe).^39,40^ However, QISP amino acids employed in ^1^H-detected ^13^C LC-photo-CIDNP require a ^13^C-label as part of the quasi-isolated spin pair, limiting the pool of cost-effective starting materials and placing a premium on simplicity and yields.^41^

Thus, we developed a straightforward chemoenzymatic route to QISP Tyr using tyrosine phenol lyase (TPL). Natively, TPL catalyzes degradation of Tyr into phenol, ammonium, and pyruvate (Figure 1B).^42,43^ This enzyme acts reversibly and, with a modest excess of one substrate, operates in the reverse synthetic direction to produce Tyr as well as Tyr analogs.^44–47^ We employed TPL from *Citrobacter freundii* (*Cfr*TPL), which undergoes high-level heterologous expression in *Escherichia coli* and has been previously employed to generate natural-abundance Tyr.^46,48^ We used 90 mM phenol and pyruvate at 0.1 mol % of catalyst relative to substrate. Importantly, we relied on excess ammonium to shift the equilibrium toward Tyr formation. Product formation using nonlabeled starting materials proceeded with >95% yield in 3 h (Figure S2).

With practical biocatalytic conditions in hand, we next tested strategies to site-specifically control deuterium incorporation. We readily produced 3,3,3-^2^H-pyruvate, (hereafter denoted as pyruvate-D_3,_ Figure S3A) via H/D exchange at pD 8.5 (Figure S3B). This material was used directly (i.e., without isolation) for a subsequent reaction with *Cfr*TPL, phenol, pyridoxal phosphate (PLP) and ND_4_Cl. In order to minimize proton content in TPL reactions, enzyme, buffer, and PLP were premixed, lyophilized and resuspended in D_2_O. These perdeuterated materials were then used to generate a Tyr isotopolog exclusively bearing deuterons at the ^13^C^α^ and C^β^ sites, as confirmed by NMR (Figure S4).

To remove the D^α^ (i.e., the D incorporated at the C^α^ site), we started by reviewing the known chemoenzymatic approaches for stereoselective C^α^-H/D exchange.^49–51^

Based on this analysis, we elected to use the PLP-dependent DsaD enzyme from *Streptomyces scopuliridis*, denoted here as DsaD, which stands out for its operational simplicity and established activity on Tyr.^49,52^ We applied the previously reported conditions to Tyr-D_3_, and observed >95% analytical yield of β,β-^2^H-Tyr (Tyr-D_2_, Figure S5). Lastly, we repeated the entire process starting from ^13^C^α^-sodium pyruvate and phenol-D_6_, to generate QISP Tyr (see Supporting Methods, and Figures 1B and S6). Characterization of QISP Tyr was carried out via UPLC mass spectrometry (UPLC-MS) and pulse-acquire ^1^H NMR. The presence of a strong ^1^H^α^ resonance at 3.91 ppm in the NMR spectrum and lack of other protons confirmed the identity of the desired product (Figure 1C left panel). Very weak signals corresponding to traces of residual nondeuterated material (3.2%) are also present in the H^β^ and aromatic (ε _1,2_ and δ_1,2_ resonances) regions.^33^ As expected, the ^13^C spectrum of QISP Tyr (Figure 1C, right panel) shows a single resonance in the C^α^ region at 56.19 ppm. This resonance converts to a doublet in the absence of ^1^H decoupling during acquisition.

In summary, QISP Tyr was produced in high yields by a simple chemoenzymatic approach. Notably, the modular nature of the synthetic route introduced here also provides access to a wide variety of other Tyr isotopologs, which are readily obtained upon simply varying the isotopic composition of starting materials and solvents (e.g., see Supporting Information).

### QISP Tyr Can Be Readily Hyperpolarized in Solution, Displaying Significant NMR Sensitivity and Prompt Detectability at 200 nM Concentration

Quasi-isolated spin pairs (QISP) are defined as two directly bonded NMR-active nuclei located within molecules whose other atoms bear mostly NMR-inactive nuclei or nuclei with low gyromagnetic ratios.^23^ We recently demonstrated that, due to the attenuation of photo-CIDNP cancellation effects resulting from multiple nearby NMR-active nuclei, small-molecules like QISP Trp (Trp-α-^13^C-β,β,2,4,5,6,7-d_7_) give rise to much higher LC-photo-CIDNP hyperpolarization than the corresponding non-QISP analogs.^23^ The QISP effect was recently further exploited in additional investigations.^20,21^ It was never, however, probed in the context of the Tyr amino acid. After preparing QISP Tyr as described in the previous section, we proceeded to perform 1D ^1^H-detected ^13^C LC-photo-CIDNP on this isotopolog at 1 μM concentration using the ^13^C RASPRINT pulse sequence. As a reference, we also carried out regular ^1^H pulse-acquire experiments lacking a hyperpolarization module. As shown in Figure 2A, the sensitivity enhancement was only moderate in the case of ^1^H pulse-acquire 1D NMR. In contrast, LC-photo-CIDNP on QISP Tyr led to 16.9-fold enhancements in sensitivity per unit concentration (Sens_C_, Figure 2B), relative to unlabeled Tyr. The Sens_C_ parameter is defined as follows

1 $${\mathrm{Sens}}_{C}=S/N/\left(\mathrm{conc}.\sqrt{t}\right)$$

where S/N, conc. and *t* denote the NMR signal-to-noise, sample concentration, and total experimental time, respectively. As expected, the observed ^1^H^α^ singlet of QISP Tyr lacks the ABX splitting of the corresponding ^1^H^α^ resonance of unlabeled Tyr (Figure 2A). The above results are quantitated in Table 1, along with additional comparisons among Sens_C_ ratios. The ATTO Thio 12 photosensitizer performs better than fluorescein, in the case of Tyr as the molecule of interest (Figure 2C and Table 1).^53^ This result is in contrast with the behavior of Trp, which displays much higher sensitivity in the presence of the fluorescein dye.^26^

**Figure 2:**
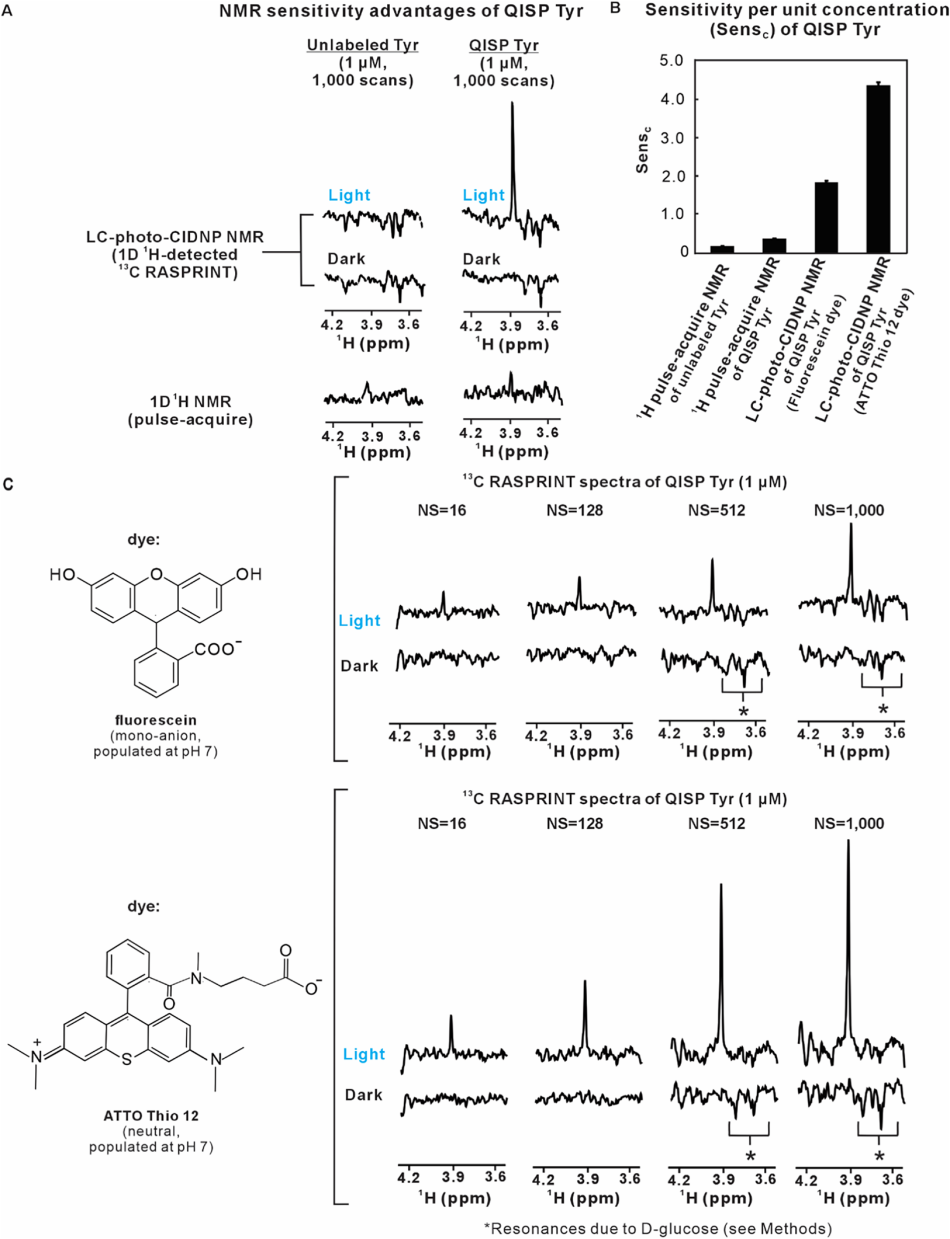
QISP Tyr enables highly sensitive NMR spectroscopy via LC-photo-CIDNP. (A) 1D ^1^H-detected ^13^C LC-photo-CIDNP and regular ^1^H NMR spectra of QISP Tyr and unlabeled Tyr in 10 mM potassium phosphate (pH 7.2) and 10% D_2_O (*n* = 2). The ^1^H^α^ spectral region is shown. LC-photo-CIDNP was performed with the ^1^H-detected 1D ^13^C RASPRINT pulse sequence under light (LED-on) and dark (LED-off) conditions in the presence of the fluorescein dye (2.5 μM). Pulse-acquire ^1^H NMR data were collected with solvent suppression (W5 with excitation sculpting) and ^13^C decoupling during acquisition (via GARP). The recycle delay of all experiments was 50 ms. The weak signals within the 3.6–3.8 ppm region of both light and dark LC-photo-CIDNP spectra are due to d-glucose (2.5 mM), which was added to the samples as part of the oxygen-scavenging procedure (see Methods and Figure S8). Note that, while the LC-photo-CIDNP of Tyr is emissive, all spectra were phased to render the Tyr ^1^H^α^ resonance positive. Consequently, the d-glucose resonances of LC-photo-CIDNP spectra appear as negative. (B) Block diagram illustrating the sensitivity per unit concentration (Sens_C_) of unlabeled and QISP Tyr (avg ± SE for *n* = 2). (C) Side-by-side comparison of LC-photo-CIDNP NMR spectra of QISP Tyr (1 μM, ^13^C RASPRINT) under light (LED-on) and dark (LED-off) conditions in the presence of either the fluorescein (top) or ATTO Thio 12 (bottom) as photosensitizer dyes (*n* = 2). The chemical structures of both photosensitizer dyes are shown on the left. Optimized concentrations of fluorescein (2.5 μM) and ATTO Thio 12 (5.0 μM) were employed, in the respective experiments. All experiments under light (LED-on) conditions were carried out with 200 ms of irradiation per scan. The experiments with fluorescein and ATTO Thio 12 were carried out with two different single-chip LED setups (UHP-mic-LED-450, a.k.a., UHP-LED-blue; Prizmatix, Holon, Israel and UHP-T-545-SR respectively) equipped with a fiber adaptor. The LED power at the fiber tip of the coaxial insert for the photoexcitation of fluorescein and ATTO Thio 12 were 0.49 W. See also Supporting Information and Supporting Figure S9 for comparisons of excitation rate constants. All data were acquired at 14.1 T (600 MHz).

**Table 1:** Direct Comparisons between NMR Sensitivity Per Unit Concentration (Sens_C_) and Sensitivity Enhancement Displayed by Unlabeled and QISP Tyr^a^

tyrosine isotopolog	Sens_C_ of pulse-acquire ^1^H NMR (no hyperpolarization)	Sens_C_ of 1D LC-photo-CIDNP NMR (^13^C RASPRINT)	sensitivity enhancement
QISP Tyr (Fluorescein dye)	0.41 ± 0.01 (A)	1.87 ± 0.04 (B)^b^	7.2 ± 0.2 (B/D)
	0.40 ± 0.02 (C)^b^		4.5 ± 0.1 (B/A)
			4.72 ± 0.05 (B/C)^b^
QISP Tyr (ATTO Thio 12 dye)	0.41 ± 0.01 (A)	4.4 ± 0.1(F)^b^	16.9 ± 0.5 (F/D)
	0.40 ± 0.02 (C)^b^		10.7 ± 0.3 (F/A)
			11.1 ± 0.5 (F/C)^b^
Unlabeled Tyrosine	0.258 ± 0.005 (D)	0	1.59 ± 0.06 (A/D)
	0.21 ± 3 × 10^–5^ (E)^b^		1.9 ± 0.1 (C/E)^b^

a All data were collected on a 14.1 T (600 MHz) NMR spectrometer and are based on NMR measurements on the Tyr ^1^H^α^ resonance. Unless otherwise stated, all experiments were performed on 1 μM (light) and 500 μM (dark or pulse-acquire) samples, with a 1.5 s recycle delay. Data are shown as avg ± SE for n = 2. See details in Methods.

b These data are based on an experiment with a 50 ms recycle delay.

The substrate-dependent performance of LC-photo-CIDNP dyes may at first appear puzzling. On the other hand, the need to have molecules of interest matched to appropriate dyes is a mere consequence of the LC-photo-CIDNP dependence upon parameters related to the identity of both dye and molecule of interest. These parameters include differences in g-factors, radical-pair lifetimes, and differences in redox potentials of photoexcited dye and molecule of interest.^22,28,29,54^ Indeed, systematic optimization of matchings between dye and molecule-of-interest in photo-CIDNP is presently the subject of active investigation.^33,35,55^ Importantly, the QISP Tyr concentration can be lowered down to nanomolar levels (200 nM), yielding prompt detectability in less than 9 min, as shown in Figure 3.

**Figure 3:**
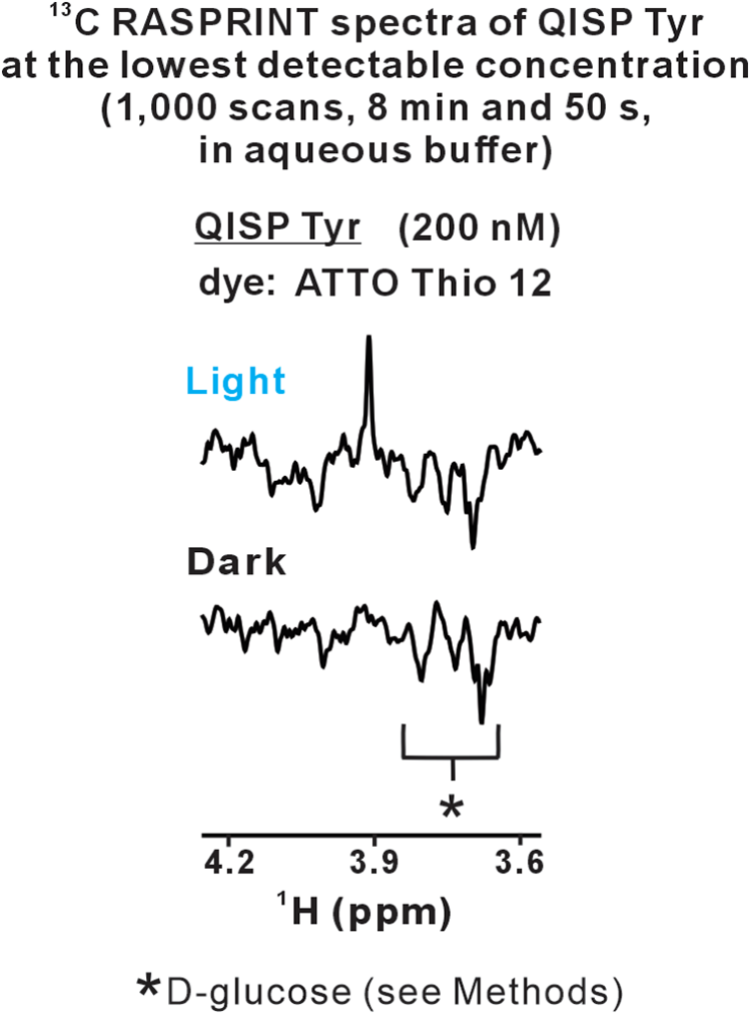
LC-photo-CIDNP enables the highly sensitive detection of Tyr in solution. LC-photo-CIDNP NMR leads to the ultrasensitive detection of 200 nM QISP Tyr (dye: ATTO Thio 12, *n* = 2) in less than 9 min. Data acquisition and processing parameters match those of Figure 2. Data were collected on a 14.1 T (600 MHz) NMR spectrometer.

In all, the above analysis shows that QISP Tyr in combination with ^1^H-detected ^13^C LC-photo-CIDNP affords much better NMR sensitivity per unit concentration (Sens_C_) than unlabeled Tyr (Figure 2B). Experiments are very fast and lead to the detection of 200 nM Tyr in aqueous solution in only a few minutes (Figure 3).

### QISP Tyr Outperforms Other Tyr Isotopologs

As shown in Figure 4 and Table 2, QISP Tyr exhibits significantly stronger LC-photo-CIDNP hyperpolarization than unlabeled Tyr, uniformly ^13^C- and ^15^N-labeled Tyr (Tyr-U–^13^C,^15^N) and ^13^C^α^ selectively labeled Tyr (Tyr-α-^13^C). The sensitivity enhancement is most prominent relative to the corresponding value for unlabeled Tyr, but it is also significant in the case of the other isotopologs (Figure 4A). The improved performance of QISP Tyr relative to both unlabeled Tyr and other ^13^C-enriched isotopologs is best assessed upon comparing LC-photo-CIDNP enhancement factors ε, defined as

$$\varepsilon =\frac{{\mathrm{area}}_{\mathrm{light}}\times {\left[\mathrm{Tyr}\right]}_{\mathrm{dark}}}{{\mathrm{area}}_{\mathrm{dark}}\times {\left[\mathrm{Tyr}\right]}_{\mathrm{light}}}$$ 2

where area and [Tyr] denote the ^1^H^α^ resonance area and Tyr concentration, respectively, under light (LED-on) and dark (LED-off) conditions. Key ε values of different Tyr isotopologs for ^13^C LC-photo-CIDNP (RASPRINT pulse sequence) are shown in Figure 4B. While the numerical values of ε are overall not very large, the improvement relative to unlabeled Tyr is significant. Note that no signal was experimentally observed under light conditions, in the case of unlabeled Tyr (Figure 4A). The high performance of QISP Tyr relative to unlabeled Tyr is ascribed primarily to the fact that the intermolecular collisions with the photoexcited triplet state of the dye are much more efficient with QISP Tyr, where the ^13^C isotope is 90.2-fold more abundant,^22,27^ favoring ^13^C LC-photo-CIDNP.

**Figure 4:**
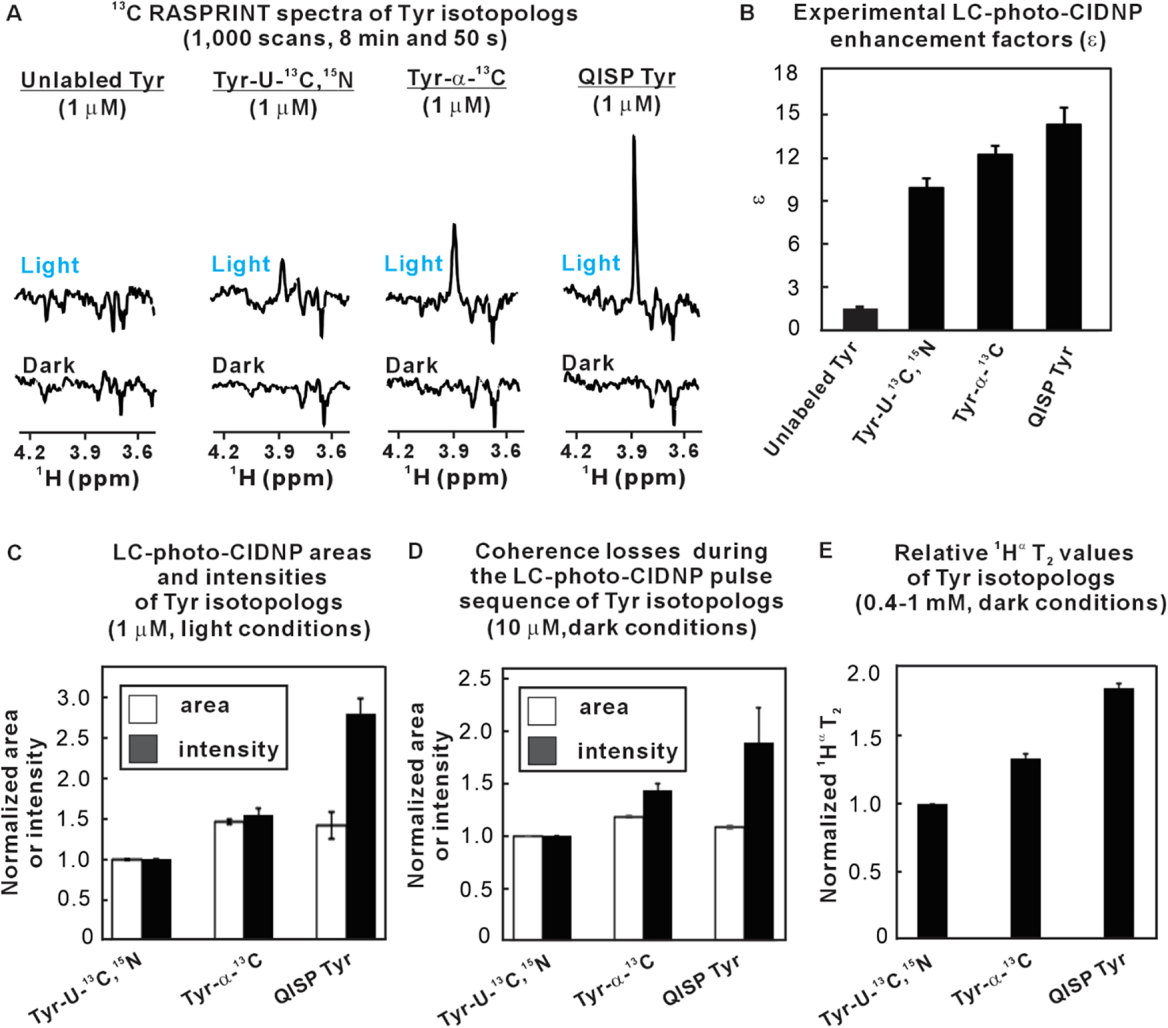
QISP Tyr outperforms its isotopologs and leads to significant ^1^H nuclear-spin hyperpolarization. (A) Side-by-side comparison between the LC-photo-CIDNP NMR spectra of several Tyr isotopologs (1 μM, ^13^C RASPRINT) under light (LED-on) and dark (LED-off) conditions (*n* = 3). Acquisition and processing parameters match those of Figure 2. (B) Experimental LC-photo-CIDNP enhancement factors (ε) of Tyr isotopologs, determined for 1 and 10 μM samples, from data under light and dark conditions (avg ± SE for *n* = 2). (C) Assessment of overall ^1^H^α^ spectral features of LC-photo-CIDNP data on Tyr isotopologs in terms of resonance areas and intensities (avg ± SE for *n* = 3). All spectra were acquired with the ^13^C RASPRINT pulse sequence under light conditions. The recycle delay was 50 ms. The total experiment time was 8 min and 50 s. (D) Graph illustrating the extent of coherence losses during the ^13^C RASPRINT pulse sequences due to a dark (LED-off) effect, assessed via changes in resonance areas. Intensity changes under dark conditions are also shown (avg ± SE for *n* = 2). (E) Graph showing the relative variations in ^1^H^α^ T_2_ of different Tyr isotopologs (avg ± SE for *n* = 2). Absolute T_2_ values can be found in Supporting Figure S7. The ^1^H^α^ nucleus of QISP Tyr has a longer T_2_ than the other isotopologs, highlighting a dark effect that contributes to the largest NMR sensitivity enhancement displayed by QISP Tyr. All data were collected on a 14.1 T (600 MHz) NMR spectrometer.

**Table 2:** NMR Sensitivity Per Unit Concentration (Sens_C_) of Three Tyr Isotopologs Bearing Variable Types of Isotopic Enrichments^a^

Tyr isotopolog	Sens_C_ of pulse-acquire ^1^H NMR (no hyperpolarization) (A)	Sens_C_ of 1D LC-photo-CIDNP NMR (^13^C RASPRINT) (B)	sensitivity enhancement(B/A)
Tyr-U–^13^C,^15^N	0.21 ± 0.01	0.71 ± 0.05	3.4 ± 0.3
Tyr-α-^13^C	0.1535 ± 0.0001	1.01 ± 0.02	6.6 ± 0.1
QISP Tyr (Fluorescein dye)	0.41 ± 0.01	1.87 ± 0.04	4.5 ± 0.1
QISP Tyr (ATTO Thio 12 dye)	0.41 ± 0.01	4.4 ± 0.1	10.7 ± 0.3

a All data are based on NMR measurements on the Tyr ^1^H^α^ resonance at 14.1 T (600 MHz). Unless otherwise stated, all experiments were performed on 1 μM (light) and 360–500 μM (dark or pulse-acquire) samples with a 50 ms and 1.5 s recycle delay respectively. Data are shown as avg ± SE for n = 2. See details in Methods.

The improved sensitivity of QISP Tyr relative to the isotopically enriched isotopologs arises from more subtle light and dark effects, which have been quantified in Table 3. First, when compared to uniformly ^13^C-labeled Tyr, QISP Tyr experiences fewer magnetization losses during the ^13^C RASPRINT pulse sequence, due to missing scalar couplings to other ^13^C or ^1^H nuclei (Figure 4D).^23^

**Table 3:** Quantitative Evaluation of Light and Dark Contributions to the Overall Sensitivity of LC-Photo-CIDNP NMR Experiments on Different Tyr Isotopologs^a^,^b^

	relative sensitivity partitioned into individual contributions				
Tyr isotopolog	LC-photo-CIDNP hyperpolarization^c^ (light effect)	elimination of coherence losses during pulse sequence (fewer *J*-couplings^d^, dark effect)	linewidth reduction^e^ (^1^H^α^ T_2_, dark effect)	product of individual contributions	overall relative sensitivity (assessed via ^13^C RASPRINT, light cond.)^f^
Tyr-U–^13^C,^15^N	100%	100%	100%	100%	100%
Tyr-α-^13^C	123 ± 10%	119 ± 1%	134 ± 4%	196 ± 17%	154 ± 8%
QISP Tyr	144 ± 15%	109 ± 1%	186 ± 4%	292 ± 31%	280 ± 19%

a Data were collected on a 14.1 T NMR spectrometer (600 MHz).

b Uniformly labeled Tyr (Tyr-U–^13^C,^15^N) is regarded as a reference compound. Unless otherwise stated, data are shown as avg ± SE for *n* = 2.

c Determined from data in Figure 4B.

d Determined from data in Figure 4D.

e Assessed from ^1^H^α^ T_2_ experiments (see Supporting Information Figure S7).

f Sensitivity values were assessed from resonance intensities of experiments run with identical parameters. Data are shown as avg ± SE for *n* = 3

A second dark effect arises from the effect of isotopic substitutions on transverse ^1^H nuclear relaxation, which in turn affects line widths at half-maximum and signal intensities. Indeed, this effect is operative, as testified by the longer ^1^H^α^ T_2_ values of QISP Tyr relative to the other isotopologs (Figure S7). The greater polarization of QISP Tyr is also contributed by light effects, which are a direct consequence of nuclear-spin hyperpolarization due to LC-photo-CIDNP. These light effects were quantified via the enhancement factors ε.^23^

In order to gain further insights into the origin of the light effects, we performed computational predictions according to Adrian,^56^ to gather information on the expected percent of geminate polarization displayed by different Tyr isotopologs, including QISP Tyr. The presence of both Tyr isotopologs and fluorescein photosensitizer in the pertinent radical pairs (Supporting Table 1) was explicitly taken into account following known procedures.^21^ The results, reported in Figure 5, show that the QISP isotopic substitution is expected to lead to large polarization enhancements at low applied field (0.47–5.9 T, i.e., ∼20–250 MHz) due to ^13^C geminate polarization. Yet, no significant enhancements are expected at 14.1 T. On the other hand, our experimental data summarized in Table 3 and Figure 4B show that QISP Tyr outperforms Tyr-U–^13^C,^15^N due to effects under light conditions, at 14.1 T. Specifically, the ratio of the photo-CIDNP-induced polarization generated by QISP Tyr over Tyr-U–^13^C,^15^N and due solely to light effects is 1.44 ± 15% (Table 3). Phenomena related to different ^13^C T_1_ relaxation times of the two isotopologs during steady-state optical irradiation (light conditions) are ruled out (see section below on experimental ^13^C T_1_ values). Hence, the experimentally observed enhanced photo-CIDNP polarization of QISP Tyr relative to Tyr-U–^13^C,^15^N under light conditions at 14.1 T is contributed by presently unknown effects occurring during either the geminate or F-pair polarization times. These effects are likely of complex origin and beyond the scope of this work.

**Figure 5:**
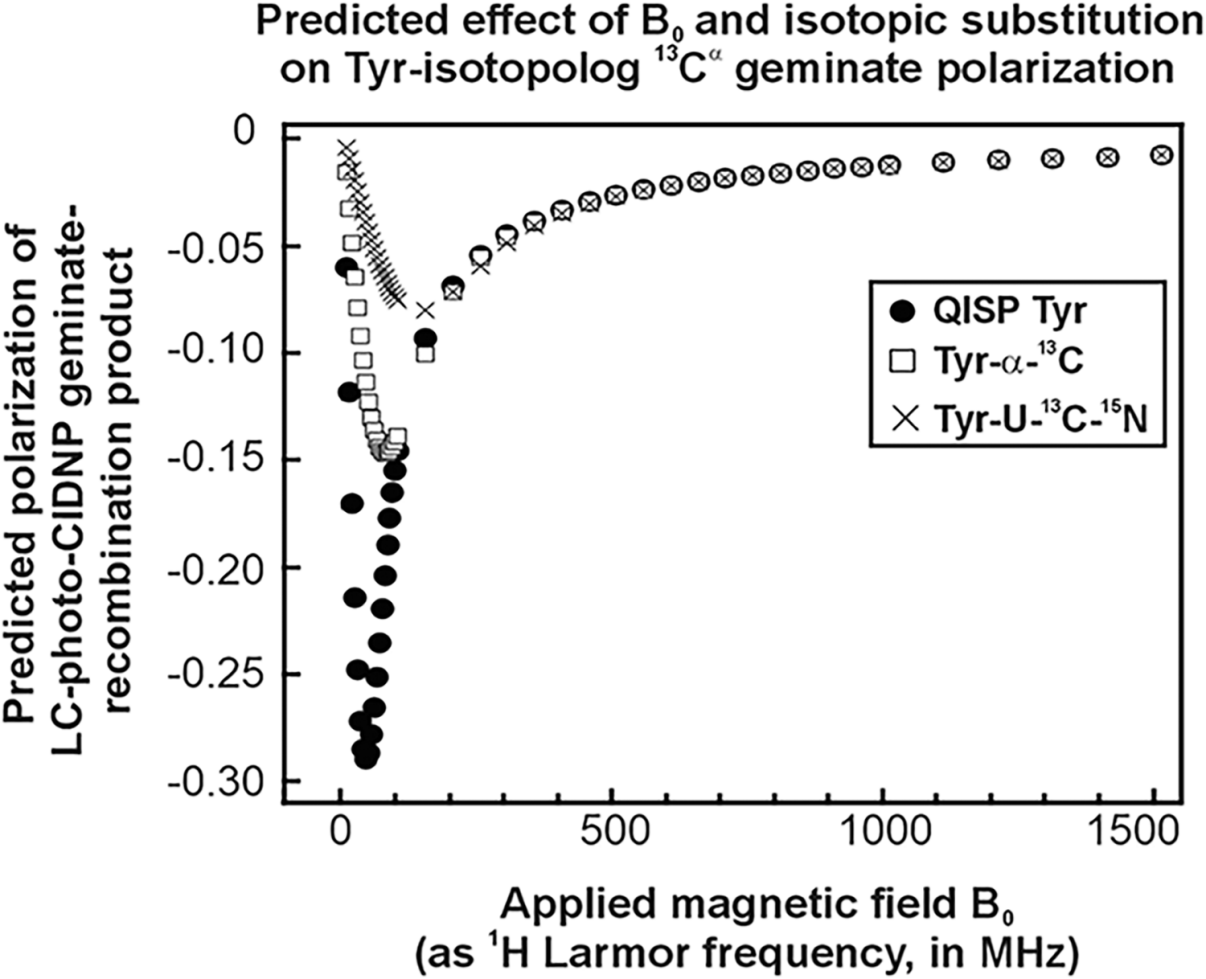
Computational predictions of ^13^C^α^ LC-photo-CIDNP geminate polarization of Tyr isotopologs highlight a large expected enhancement at low magnetic field. Computational predictions of LC-photo-CIDNP geminate polarization of ^13^C^α^ for Tyr-U–^13^C,^15^N, Tyr-α-^13^C and QISP Tyr as a function of applied magnetic field. The simulations were performed upon taking into account the known g-factors and hyperfine coupling constants of the TyrO^•^ radical and the fluorescein anion radical (Fl^•‑^) (see details in Supporting Information).

In summary, the above arguments show that our results on QISP Tyr at 14.1 T (relative to Tyr-U–^13^C,^15^N) at 14.1 are due to the combined effect of (a) photo-CIDNP independent dark effects, including increases in ^1^H T_2_ values during acquisition leading to line width reduction and reduced coherence losses during the pulse sequence (see Figure 5 and Table 3), and (b) steady-state photo-CIDNP ^13^C light effects that are independent of ^13^C T_1_ relaxation during optical irradiation.

Finally, as shown in Table 3, the product of the individual experimental dark and light contributions is in good agreement with the overall relative sensitivities assessed experimentally via ^13^C RASPRINT experiments at 600 MHz, in support the above analysis and consistent with previous assessments on QISP Trp.^23^

### QISP Tyr Performs Even Better at Lower Magnetic Field

Encouraged by the computational results in Figure 5, which predict a much larger photo-CIDNP geminate polarization for QISP Tyr at lower applied field than 14.1 T (600 MHz), we also performed experiments on a 1.88 T (80 MHz) benchtop NMR spectrometer. The results are shown in Figure 6. Indeed, at 80 MHz QISP Tyr displays a substantially higher enhancement factor (ε) of ∼313, relative to ∼30 for Tyr-U–^13^C,^15^N. This value corresponds to a ∼10.4-fold better performance. These data provide a further improvement relative to the 1.44-fold increase originally observed at 14.1 T (600 MHz). The stark difference between QISP Tyr and Tyr-U–^13^C,^15^N at low-field, coupled with the theoretical predictions of Figure 5, strongly suggest that the QISP Tyr isotopolog leads to superior LC-photo-CIDNP performance at low field, due to the increased extent of geminate polarization due to the “QISP effect”.

**Figure 6:**
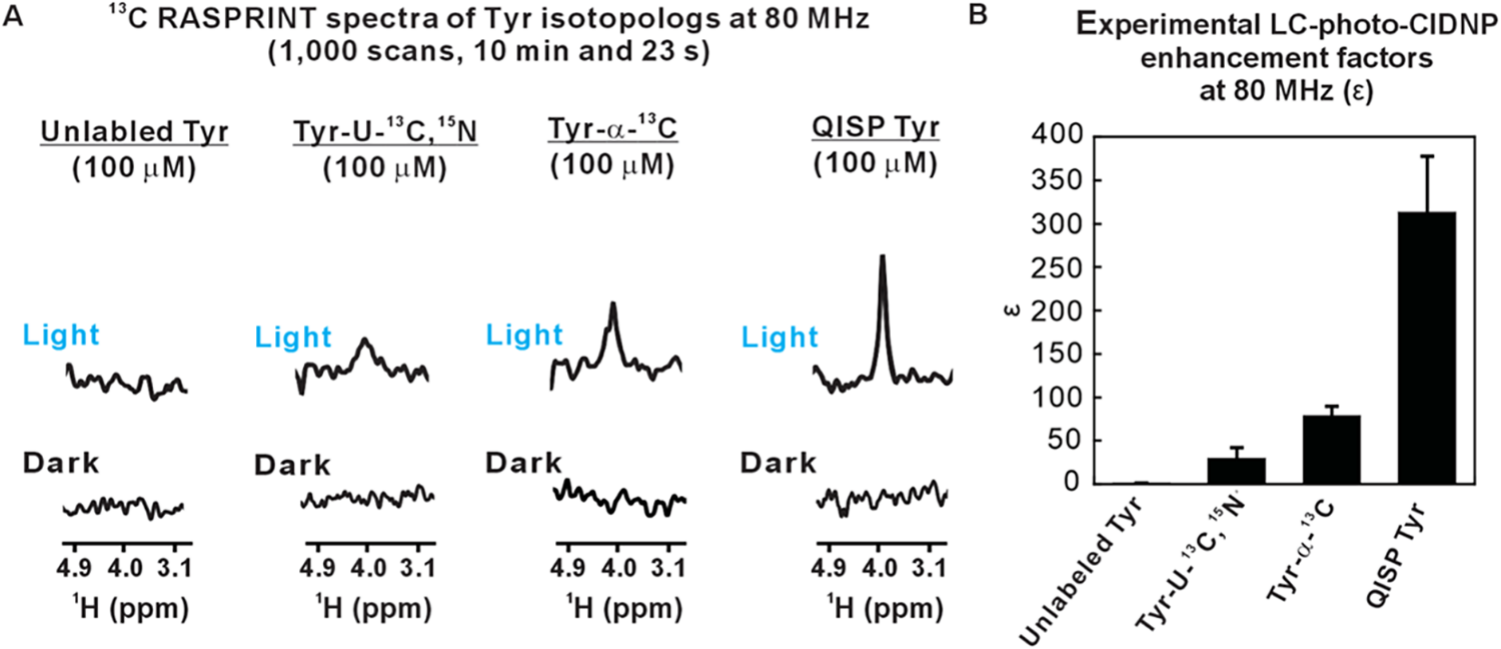
QISP Tyr outperforms other isotopologs and exhibits large LC-photo-CIDNP enhancement factors at low field (80 MHz, benchtop NMR spectrometer). (A) Side by side LC-photo-CIDNP spectra of various Tyr isotopologs (100 μM, ^13^C RASPRINT). All data were collected under light (LED-on) and dark (LED-off) conditions at low applied field (1.88 T, 80 MHz, *n* = 2). (B) Experimental LC-photo-CIDNP enhancement factors (ε) of Tyr isotopologs, assessed on 100 μM samples (256 scans) from data under light and dark conditions (avg ± SE for *n* = 2). Data were acquired with a 0.05 s recycle delay and 0.2 s LED irradiation per scan, using 25 μM fluorescein as photosensitizer. Processing parameters and experimental setup are identical to those of Figure 2A.

### T_1_ Effects Do Not Contribute to the Observed ^13^C Hyperpolarization

To test the potential effects of differential longitudinal relaxation (T_1_) during our steady-state ^13^C LC-photo-CIDNP irradiation, we performed ^13^C^α^ -T_1_ measurements on all three isotopologs both at high (14.1 T, 600 MHz, *n* = 3) and low (1.88 T, 80 MHz, *n* = 2) magnetic field. We found that, at each of these applied fields, the T_1_ of the various isotopologs are statistically indistinguishable from each other (Supporting Figure S8). As a result of these measurements, we conclude that different T_1_ values are not responsible for the different steady-state LC-photo-CIDNP outcomes at both 14.1 and 1.88 T.

### QISP Tyr Can Be Readily Detected in Complex Physiologically Relevant Media

Next, we tested whether the LC-photo-CIDNP sensitivity advantage of QISP Tyr can also be extended to complex physiologically relevant media. We focused on an in-house prepared (Figure S11) bacterial cell extract, following a protocol similar to the one by Bakke et al.^57^ This extract contains thousands of proteins,^58^ chaperones^58,59^ and small molecules, including machinery devoted to amino-acid-biosynthesis and processing. As shown in Figure 7 (see also Figure S12), the ^1^H^α^ resonance of QISP Tyr (10 μM) spiked in this medium reproducibly displays a ca. 2-fold intensity enhancement relative to dark conditions. Data were collected fast, in less than 9 min. This result confirms that the QISP/LC-photo-CIDNP approach works in complex milieux, despite the somewhat attenuated sensitivity advantage relative to simple aqueous buffer.

**Figure 7:**
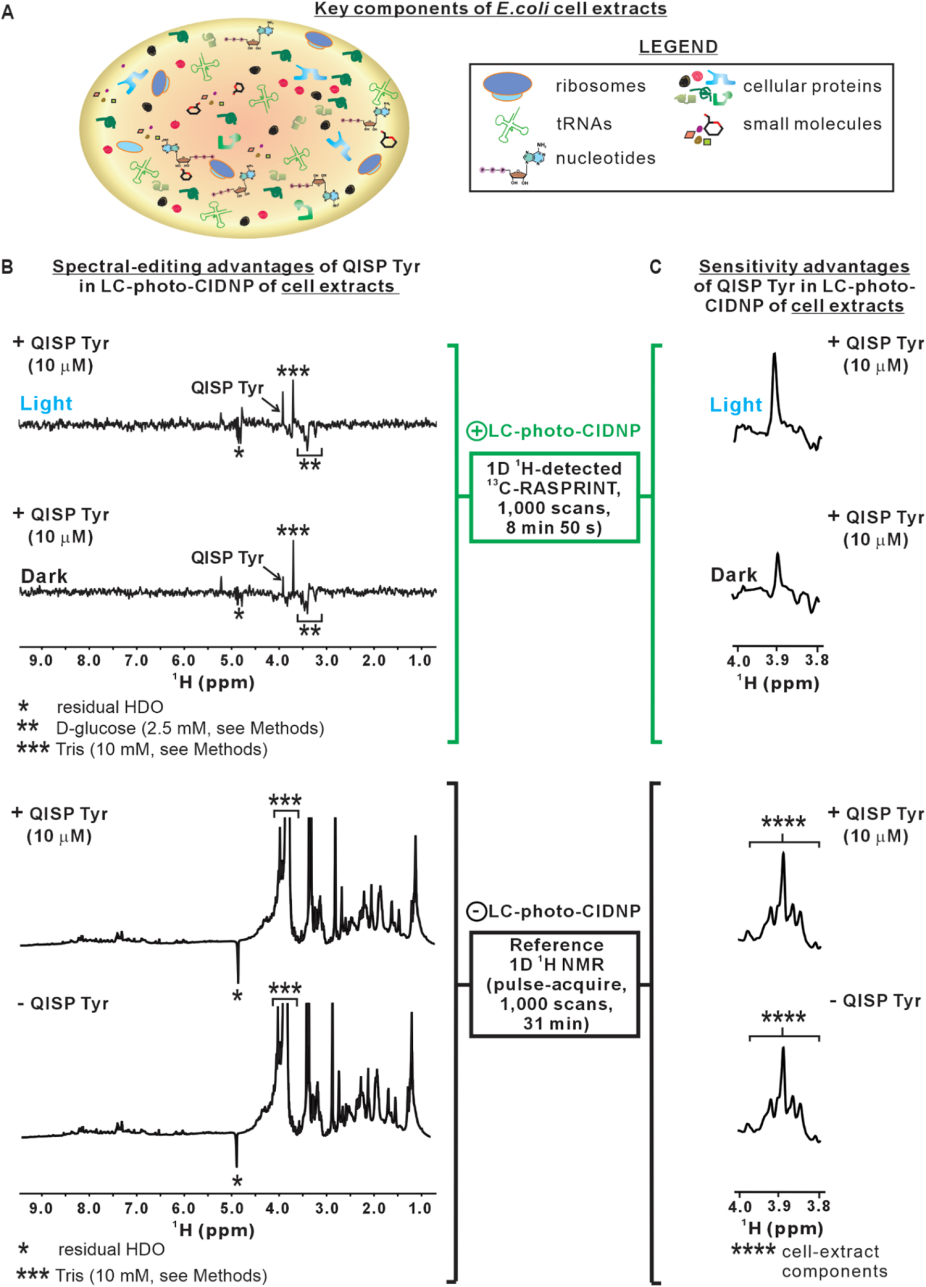
LC-photo-CIDNP in combination with selective isotope labeling enables data collection in cell extracts. (A) Schematic illustration of the main components of the bacterial cell extract employed in this work. (B) *Top-two spectra*: LC-photo-CIDNP NMR spectra of 10 μM QISP Tyr added to an in-house-prepared *E. coli* cell extract (5-fold diluted, pH 7.3) in the presence of 10% D_2_O. Data were collected under both dark (i.e., LED-off) and light (i.e., LED-on) conditions (*n* = 2). *Bottom-two spectra*: reference pulse-acquire ^1^H NMR spectra (no-photo-CIDNP) of an in-house prepared *E. coli* cell extract (5-fold diluted, pH 7.4) in the absence (0 μM) or presence (10 μM) of QISP Tyr (*n* = 2). (C) Close-up views of the ^1^H^α^ region of the LC-photo-CIDNP (top) and ^1^H pulse–acquire (bottom) spectra in panel B. Solvent suppression was achieved by W5 excitation sculpting, and ^13^C decoupling during acquisition was achieved via GARP. The recycle delays of the LC-photo-CIDNP and pulse-acquire ^1^H NMR experiments were 50 ms and 1.5 s, respectively. The LED irradiation time per scan was 0.2 s (replaced by a 0.2 s LED-off delay under dark conditions). The resonances denoted as **** are due to unknown endogenous components of the cell extract. All data were acquired on a 14.1 T­(600 MHz) NMR spectrometer.

Control LC-photo-CIDNP experiments in cell extracts performed in the absence of Tyr spiking displayed no resonance at 3.9 ppm, in either the dark or light spectra (data not shown). This result confirms the assignment of the sharp resonance at 3.9 ppm to the ^1^H^α^ of QISP Tyr. Interestingly, this QISP-Tyr resonance also shows up under dark conditions, though with lower intensity. This is due to the fact that, as discussed in the previous section, the QISP NMR-sensitivity advantage includes effects under both light and dark conditions. Therefore, even dark spectra are expected to yield excellent detectability. Control 1D ^1^H pulse-acquire experiments (lacking any photo-CIDNP) showed that a ^1^H resonance at 3.9 ppm, nearly overlapping with that of QISP Tyr, is also present in cell extracts that have not been spiked with QISP Tyr (Figures 7B and S12B). Identical set of resonances in the absence and presence of QISP Tyr suggest that this amino acid is not detectable by regular pulse-acquire 1D ^1^H NMR. These ^1^H pulse-acquire NMR resonances are due to endogenous components of the bacterial cell extract. Conveniently, the ^13^C-RASPRINT data of Figure 7B (two upper spectra) do not show any of these background resonances because the ^13^C RASPRINT experiment selects molecules that contain ^13^C followed by ^1^H detection via reverse INEPT. Indeed, the cell extract does not endogenously contain any ^13^C-enriched materials, thus abrogating detection of thousands of cell-milieu components, and thus considerably “cleaning-up” the entire ^13^C RASPRINT LC-photo-CIDNP spectral width (see also Figure S12).

Importantly, the disease- and metabolism-relevant concentrations of Tyr in serum^60^ and brain^61^ fall within the low-μM range or higher. In other words, they correspond to levels that are readily detectable by LC-photo-CIDNP according to the data of Figure 7. In all, the above data highlight the promise of the QISP/LC-photo-CIDNP approach for the direct in situ detection of tyrosine metabolism in complex physiological media.

### Natural-Abundance Tyr and Neurotransmitters are Detected at Extremely Low Concentration, Down to 10 nM, via ^1^H LC-Photo-CIDNP

Next, we decided to test natural-abundance Tyr and its catabolites epinephrine and L-DOPA. The idea behind these experiments is to broaden the scope of LC-photo-CIDNP characterization to scenarios where spiking with QISP molecules is neither possible nor helpful. Under these circumstances, direct detection of Tyr and its metabolic products, especially if performed *in situ* under nonperturbing conditions, was previously unattainable. Yet, it is highly desirable.

As shown in Figure 8A, a 1D pulse-acquire NMR pulse sequence including ^1^H LC-photo-CIDNP LED-irradiation and solvent suppression (^1^H PASS-W5es) was employed in aqueous buffer to explore the detectability of natural-abundance Tyr, epinephrine and L-DOPA. As shown in Figure 8B–D, not only unlabeled Tyr produced intense resonances at 1 μM concentration under light conditions, but also the two Tyr catabolites. In the specific case of epinephrine, a strikingly intense signal was detected for the methyl resonance at 1 μM concentration. The spectral feature for epinephrine was so prominent that we had to employ a 40-fold vertical-scale attenuation to fully visualize the spectrum (Figure 8B).

**Figure 8:**
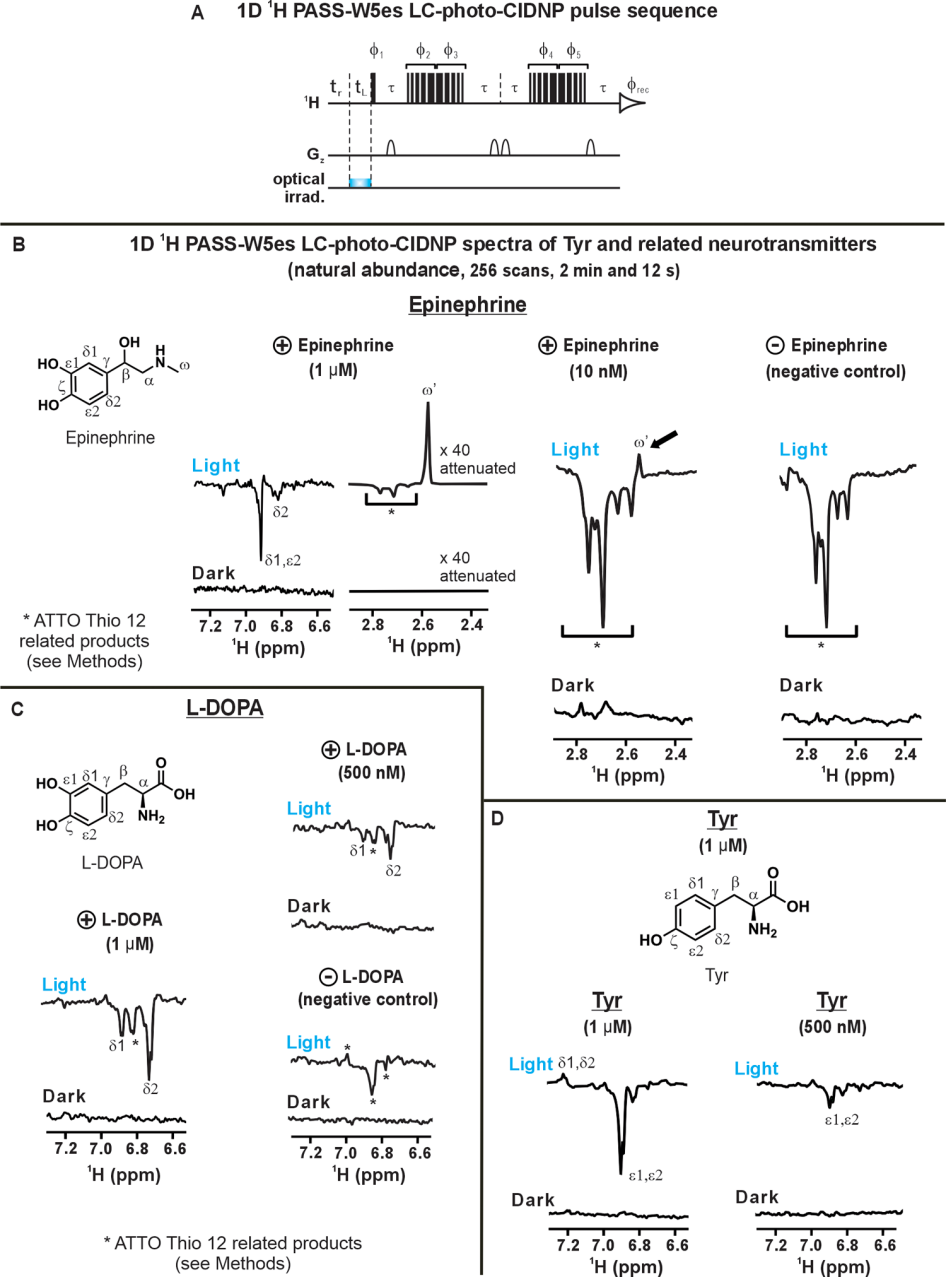
Tyr and Tyr-related neurotransmitters are readily detected by 1D ^1^H LC-photo-CIDNP at extremely low (low-nM to μM) concentration. (A) ^1^H PASS-W5es LC-photo-CIDNP pulse sequence employed for the experiments shown in this figure. The t_r_ and t_L_ symbols denote recycle delay and LED irradiation time, respectively. Solvent suppression was carried out by WATERGATE W5 excitation sculpting. (B) ^1^H PASS-W5es LC-photo-CIDNP spectra of natural-abundance epinephrine hydrochloride (racemic mixture) at 1 μM (*n* = 2) and 10 nM (*n* = 3) concentrations. The vertical scale of the 1 μM epinephrine hydrochloride spectrum (aliphatic region) was attenuated 40-fold. A negative-control experiment lacking epinephrine confirmed that the absorptive peak at 2.58 ppm (highlighted by the arrow) corresponds to a photoproduct of the the epinephrine hydrochloride methyl resonance (denoted as ω', see Supporting Figures S14 and S15). Strong emissive peaks in the aliphatic region arise from ATTO Thio 12 photoreaction products (see Methods and Supporting Figure S14). (C) ^1^H PASS-W5es LC-photo-CIDNP spectra (aromatic region) of natural-abundance L-DOPA (1 μM and 500 nM; *n* = 2). A negative-control experiment lacking L-DOPA (below, right; *n* = 2) confirmed the emissive peak ∼6.83 ppm corresponded to ATTO Thio 12 related products undergoing photo-CIDNP. (D) ^1^H PASS-W5es LC-photo-CIDNP spectra (aromatic region) of natural-abundance Tyr (1 μM and 500 nM, *n* = 2). All experiments used the ATTO Thio 12 as a dye (5 μM) and included a 0.05 s recycle delay and a 0.2 s LED optical irradiation time (replaced by a 0.2 s LED-off delay, under dark conditions). All data in this figure were collected in 90% H_2_O containing 10 mM potassium phosphate (pH 7.2) and 10% D_2_O. All data were collected on a 14.1 T (600 MHz) NMR spectrometer.

Encouraged by the above result, we proceeded to further lower sample concentration, and we managed to get epinephrine detectability at an unprecedented 10 nM concentration in just a little more than 2 min (Figure 8B). This result most clearly outlines the power of the ^1^H LC-photo-CIDNP approach. It is significant because it represents the lowest ever detected concentration by NMR spectroscopy in solution, to the best of our knowledge.

Notably, ^19^F photo-CIDNP work by Aldrik and co-workers, employing microcoils and continuous flow to regularly replenish the dye, was able to detect a remarkably small amount of nucleotide in solution, namely 0.8 picomoles.^62^ On the other hand, this amount corresponds to an 800 nM concentration, which is significantly higher than the concentration detected in this work.

While the ^1^H^α^ protons of epinephrine also exhibited enhancement, the signal-to-noise ratio was ∼4.5-fold lower than the one due to the ^1^H methyl resonance (labeled as ω', see Supporting Figure S15B). Aromatic protons were clearly detected for L-DOPA at 500 nM, but no aliphatic protons were observed. Similarly, aromatic protons were clearly detected for Tyr at 500 nM, with weaker enhancements in the aliphatic region (data not shown).

## Discussion

### Overview

Several methodologies implementing the low-concentration (LC) version of photo-CIDNP as well as the QISP ^13^C–^1^H labeling strategies for the Trp amino acid have already been published over the past few years. The present work introduces additional new methodologies as well as new cutting-edge applications. Namely, this work focuses on the Tyr amino acid, not on Trp, and it makes significant unprecedented strides on the detectability of Tyr and its metabolites in biologically relevant contexts. This study also introduces a novel bioenzymatic synthetic strategy for the QISP Tyr isotopolog. The entirely novel photo-CIDNP applications of QISP Tyr shown here lead to its straightforward detection on a 600 MHz NMR spectrometer at 200 nM concentration. This result is significant because it raises the detection limit of Tyr above that of other Tyr isotopologs. Further, Tyr metabolites like epinephrine were readily detected at an unprecedented 10 nM level. The new synthetic methodology displays both technical innovation and clinical potential, given that Tyr plays a crucial role in multiple metabolic pathways, and that its concentration in biological fluids is frequently altered under pathological conditions. In summary, the combination of the new synthetic methodology and hypersensitive detection of Tyr and its metabolites, shown in this work, holds unique promise for both mechanistic studies and clinical applications.

### Choice of Photosensitizer Dyes

The photophysical properties of the fluorescein (Fl) and ATTO Thio 12 dyes used in this work were previously characterized.^26,53^ Based on these parameters, fluorescein (Fl) was initially expected to be an adequate sensitizer for Tyr and catecholamine, mainly due to its longer triplet-state lifetime (∼20 ms) than ATTO Thio 12 (AT12, ∼2 μs) and due to its capability of undergoing prolonged optical irradiation. Further, previous kinetic simulation and experimental data showed that, at extremely low concentration of NMR-active molecules (e.g., Tyr and Trp at ca. 1–10 μM), fluorescein performs well.^26^ Indeed, fluorescein remains the best LC-photo-CIDNP dye for the Trp amino acid, to date. On the other hand, we found that ATTO Thio 12 performs even better than fluorescein, for Tyr and catecholamines. This result is consistent with previous reports^53^ and justifications based on π-stacking and nonpolar-content arguments, as described.^63^ Therefore, ATTO Thio 12 was chosen for many of the experiments reported in this work.

### Sensitive Detection of Tyr and Neurotransmitters

In this study, we introduced novel strategies to detect the Tyr amino acid and some of its metabolites at high sensitivity and atomic resolution. This effort led to the detection of Tyr isotopologs and neurotransmitters (epinephrine and L-DOPA) at μM and sub-μM concentrations. Notably, epinephrine was detected very rapidly and efficiently at the remarkably extremely low 10 nM concentration. Given the standard liquid-state sample volumes (600–700 μL in NMR tubes of 5 mm Ø) employed in all our experiments, this value corresponds to only 1.3 nanograms. This is an extremely small amount. Further, 10 nM is the lowest concentration detected by NMR spectroscopy in liquids, to the best of our knowledge. In order to increase the *in situ* detectability of Tyr and its metabolites by liquid-state NMR, we employed two distinct approaches.

First, we generated a Tyr isotopolog bearing a quasi-isolated spin pair (QISP) via a new biosynthetic route. The resulting QISP Tyr was then used in optically enhanced NMR (^1^H-detected ^13^C LC-photo-CIDNP), upon taking advantage of the reduction of photo-CIDNP cancellation effects within QISPs. Importantly, the modular nature of the synthetic procedure employed to make QISP Tyr enables its facile extension to many other Tyr isotopologs bearing or lacking QISPs. Importantly, this strategy was shown to work in cell-like media, where a convenient large degree of spectral editing was observed (Figures 7B and Supporting Figure S12). Hence, this approach is a powerful tool to follow the catabolic fate of Tyr metabolites like catecholamines, whose structure and concentration are tightly connected to a variety of deadly disorders. A limitation of our work is that we have not yet extended our approach to the analysis of more complex media of eukaryotic origin (e.g., blood serum, cerebrospinal fluids, urine). On the other hand, the data collected here suggest that this extension is entirely possible, and we plan to pursue this exciting direction in the near future.

Second, we also explored a much more operationally simple strategy based upon employing Tyr and Tyr-related neurotransmitters at natural abundance, followed by ^1^H LC-photo-CIDNP. In this way (a) it is not necessary to perform organic synthesis, given that all the natural-abundance materials are readily commercially available, and (b) this approach enables the straightforward *in situ* analysis of naturally occurring metabolites that are already present in the medium of interest. While in this case the detectability of Tyr is not quite as impressive as in the case of the QISP Tyr approach discussed in the previous paragraph, this strategy opens up the opportunity to detect Tyr metabolites away from the context of Tyr catabolism. In addition, this approach revealed that epinephrine can be detected at extremely low concentrations (10 nM) via ^1^H LC-photo-CIDNP.

### Outstanding Challenges

LC-photo-CIDNP of isotopically enriched molecules within complex biological matrices enables highly effective analysis at concurrently increased sensitivity and selectivity. The reason why sensitivity is coupled to selectivity is the fact that (a) only quasi-isolated spin pairs (QISPs) experience sensitivity enhancement, and (b) the RASPRINT pulse sequence selects all ^13^C–^1^H spin pairs, yet the concentration of these pairs is really low in natural-abundance complex media. In our case, QISP Tyr bears a quasi-isolated ^13^C–^1^H enrichment, which is unique among all mixture components. As a consequence, only low-concentration (down to 200 nM) QISP Tyr is detected via ^13^C RASPRINT, together with some selected and unavoidable nonspectrally overlapping buffer and glucose components, which are present at much higher concentration (mM), as shown in Figure 7. The spectral-selectivity advantage of LC-photo-CIDNP of QISP Tyr, together with the increased sensitivity for the detection of target molecules, highlights the suitability of this technology for applications targeting the analysis of aromatic-containing metabolites and their anabolic and catabolic products in complex biological media.

In general, the photo-CIDNP methodology is well-known to involve the generation of transient radicals and radical pairs, whose typical lifetimes are up to hundreds of picoseconds (geminate radical pairs) all the way up to hundreds of microseconds (F-pairs and free radicals). While these radicals are likely to experience collisions with the many complex components of physiological environments, a control experiment (Supporting Figure S13) shows that there is no detectable photodamage to cellular components. Specifically, Supporting Figure S13 shows that there is no variation in the quality of spectral features before and after extensive photoirradiation under identical conditions to those employed in the experiments of Figure 7. An additional important consideration is the fact that the most dangerous radical in both simple aqueous solution and complex biological media is expected to be singlet oxygen, due to its very high reactivity. The latter is notoriously generated upon reaction of ground-state molecular oxygen with photoexcited photo-CIDNP dyes. On the other hand, all our photo-CIDNP experiments, including those in complex cell-like media, include the biocompatible enzymes glucose oxidase (GO) and catalase (CAT), which serve as oxygen scavengers as described.^26,64^ Therefore, given the extremely low oxygen concentration in all of our samples, including those in biological media, and given the control experiment in Supporting Figure S13, photodegradation in biological media does not seem to be an additional challenge in LC-photo-CIDNP. In addition, analysis of the long-term photodegradation of Tyr and catecholamines in both buffered solutions and biological media was not analyzed in this work. Yet, its future comprehension will enable experimental optimizations a more rigorous control of the experimental conditions.

Finally, the highly desirable generalization of LC-photo-CIDNP to a wider variety of molecules requires a thorough understanding of the underlying photochemical mechanisms and spin-dynamics. While a full mechanistic interpretation and understanding of the underlying spin dynamics is a complex goal, the main parameters that need to be better controlled and mastered are the hyperfine coupling constants of photo-CIDNP dyes, the redox potentials and EPR g-factors of dyes and molecules of interest, the lifetime of the radical pair cage and the electron-transfer and back-electron transfer rate constants.

### Outlook

In summary, the strategies developed in this study highlight enticing opportunities for the detection of hydroxyphenyl-containing neurotransmitters at high sensitivity and resolution in their natural environment. These technologies are particularly attractive because they bypass the need to perform ionization, conversion to the gas phase, analytical purification or any other type of laboratory handling. Finally, the optically enhanced LC-photo-CIDNP NMR approach described here has the potential to be extended to metabolomic studies,^65^ and to investigations employing low-cost/low-maintenance benchtop NMR spectrometers. The latter are emerging tools in photo-CIDNP^34^ and can be easily housed in analytical laboratories, suggesting future opportunities to perform ultrarapid analysis of Tyr and Tyr-related neurotransmitters within clinical settings.

## Conclusions

In this work, we demonstrated a versatile chemoenzymatic route for the synthesis of Tyr isotopologs, including QISP Tyr, that were then employed to increase NMR sensitivity in solution via optically enhanced NMR (LC-photo-CIDNP). Given that Tyr is a key metabolite and that LC-photo-CIDNP works well in both buffered media and cell-like environments, the advances highlighted in this work bear promise to lead the way to highly efficient detection of Tyr metabolites *in situ*, in cell-like or extracellular media. The detection of epinephrine (a.k.a. adrenalin) at 10 nM concentration was particularly noticeable. In all, this work illustrates (a) the versatility of PLP-dependent enzymes to generate specifically labeled amino-acid isotopologs, and (b) the ability of LC-photo-CIDNP to achieve ultrasensitive atomic-resolution detection of Tyr and Tyr-related neurotransmitters. Both achievements, considered either in isolation or synergistically, are expected to enhance the available toolkit for the detection and analysis of biomedically relevant aromatic molecules in both basic-science and clinical settings.

## Supplementary Material



## Data Availability

All the data necessary to evaluate the conclusions of this work are provided in the article and Supporting Information. Spectral data and plasmids pertaining to this work can be obtained from the corresponding authors upon reasonable request.

## Notes

Description of synthesis and characterization of Tyr isotopologs, DNA and protein sequences, LC-photo-CIDNP experimental details on sample preparation and NMR data collection and processing under LED-on and LED-off conditions, assessment of light and dark effects used to generate Table 3, experimental details on spin–lattice (T_1_) and transverse (T_2_) relaxation data collection and processing, evaluation of optical-excitation rate constants (PDF)

## §

Thermo Fisher, 8551 Research Way, Middleton, Wisconsin 53562, United States
