# Supplementary material for: Highly Sensitive Detection of Tyrosine and Neurotransmitters by Stereoselective Biosynthesis and Photochemically Induced Dynamic Nuclear Polarization
Source: J Am Chem Soc. 2025 Nov 5;147(46):42361–74. doi: 10.1021/jacs.5c11334 (PMC12636019; doi:10.1021/jacs.5c11334)
Supplement: Supplementary file 1 [file ja5c11334_si_001.pdf]

Supplementary Information

Highly Sensitive Detection of Tyrosine and Neurotransmitters by Stereoselective Biosynthesis and Photochemically Induced Dynamic Nuclear Polarization

Ummay Mahfuza Shapla,[1,2,3] Jamorious L. Smith,[1,3] Anubhab Halder,[1] Lillian Thompson,[1] Andrew R. Buller,[1,*] Silvia Cavagnero[1,*]

[1] *Department of Chemistry, University of Wisconsin-Madison, 1101 University Ave, Madison, WI 53706*

[2] *Biophysics Program, University of Wisconsin-Madison, WI 53706*

[3]Equal contributors.

*Corresponding authors (arbuller@wisc.edu; cavagnero@chem.wisc.edu)

Ummay Mahfuza Shapla and Jamorious L. Smith contributed equally to this work. Andrew R. Buller and Silvia Cavagnero are the corresponding authors.

Materials and Methods

Materials. All chemicals and reagents used for the synthesis of QISP Tyr were purchased from commercial suppliers (Sigma-Aldrich, VWR) at the highest quality available and used without further purification unless otherwise stated. Genes were purchased as gBlocks from Integrated DNA Technologies (IDT). Unlabeled Tyr was purchased from Advanced ChemTech while Tyr-U-^{13}C,^{15}N and Tyr-α-^{13}C were purchased from Cambridge Isotope Laboratories as freeze-dried powders. Sodium pyruvate (^{13}Cα, 99%) was purchased from Cambridge Isotope Laboratories. The photosensitizer dye fluorescein (sodium salt) and ATTO Thio 12 were purchased from MilliporeSigma and ATTO-TEC GmbH respectfully. The oxygen-scavenging enzymes *Aspergillus niger* glucose oxidase (GO, Enzyme commission classification code EC 1.1.3.4) and bovine liver catalase (CAT, EC 1.11.1.6) were purchased from MilliporeSigma in freeze-dried form. D-glucose (natural abundance) and D-glucose-D_{12} were purchased from RPI (Research Product International) and Santa Cruz Biotechnology, respectively. Epinephrine hydrochloride (racemic mixture) and 3,4-Dihydroxy-L-Phenylalanine (L-DOPA) were purchased from Sigma-Aldrich.

Instrumentation employed for synthesis and characterization of QISP Tyr. *E. coli* cells were electroporated with an Eppendorf E-porator at 2500 V. New Brunswick I26R shaker incubators (Eppendorf) were used for cell growth. Cell disruption via sonication was performed with a Sonic Dismembrator 550 (Fisher Scientific) sonicator. UV-vis spectroscopic measurements were made on a UV-2600 Shimadzu spectrophotometer. Optical density measurements were collected using an Ultraspec 10 Cell density meter (Amersham Biosciences). Ultra-performance liquid chromatography-mass spectrometry (UPLC-MS) data were collected on an Acquity UPLC (Waters) equipped with an Acquity PDA and QDA MS detector using either an Intrada Amino Acid column (Imtakt) or a BEH C18 column (Waters). Preparative-scale flash chromatography

was performed on an Isolera One Flash Purification system (Biotage). NMR data were collected on Bruker 400, 500 or 600 MHz spectrometers equipped with BBFO and DCH and TCI-F cryogenic probes, respectively. The 80 MHz NMR experiments were performed on a Fourier-80 Benchtop NMR equipped with [^1H/^{13}C/^{19}F] room temperature probe and external lock. All NMR chemical shifts were referenced either to the residual solvent resonance or to the tetramethylsilane (TMS) internal standard. TMS was used for data collected in organic solvents (except for dimethyl sulfoxide, DMSO). Spectra recorded using DMSO-D_6 were referenced to the residual DMSO signal at 2.5 ppm for ^1H and 39.52 ppm for ^{13}C NMR. Spectra acquired in aqueous buffer were referenced to deuterated sodium trimethylsilylpropanesulfonate (DSS-D_6), used as external standard. NMR resonance abbreviations were s, d, t, q, dd, br, m, app denoting singlet, doublet, triplet, quartet, doublet of doublets, broad, multiplet, and apparent, respectively. High-resolution mass spectrometry data were collected with a Q Extractive Plus Orbitrap (NIH 1S10OD020022-1) instrument with electrospray ionization (ESI).

Cloning of wild-type *Cfr*TPL gene. A codon-optimized copy of the *Citrobacter freundii* tyrosine-phenol lyase (*Cfr*TPL) gene (Appendix C) was purchased as a gBlock from Integrated DNA Technologies. This DNA fragment was inserted into a pET-22b(+) vector via Gibson Assembly [1] and transformed by electroporation into *E. coli* BL21(DE3). Cells after a 30-minute recovery period in Luria-Bertani (LB) media, cells were plated onto LB plates containing 100 µg/mL ampicillin (LB_{amp}) and incubated overnight. A single colony was then used to inoculate 5 mL of Terrific Broth II medium containing 100 µg/mL ampicillin (TB_{amp}). The culture was then incubated overnight at 37 °C with shaking at 200 rpm. Next, 900 µL of the saturated cell culture were mixed with 900 µL of sterile 50% glycerol and flash-frozen in $N_{2(\ell)}$ to generate a glycerol stock.

Cloning of wild-type DsaD gene. The plasmid containing the *Streptomyces scopuliridis* DsaD gene (see Appendix C) was purchased as a construct in a pET-28b(+) vector from Twist Biosciences. This construct was transformed via standard heat-shock protocols into chemically competent *E. coli* BL21(DE3). After a 30-min recovery period in LB medium, cells were plated onto LB plates containing 50 µg/mL kanamycin (LB_{kan}) and incubated overnight. A single colony was then used to inoculate 5 mL of Terrific Broth II medium containing 50 µg/mL kanamycin (TB_{kan}). The culture was then incubated overnight at 37 °C with shaking at 200 rpm. 900 µL of the saturated cell culture were mixed with 900 µL of sterile 80% glycerol and flash-frozen in $N_{2(\ell)}$ to generate a glycerol stock.

Plasmid isolation. A 5-mL culture of *E. coli* harboring the plasmid of interest was grown overnight at 37°C with shaking at 200 rpm. The plasmid was isolated and purified using Zymo Plasmid Miniprep kits and sequenced by Functional Biosciences (Madison, WI).

Expression, purification and characterization of *Cfr*TPL. *Expression.* An overnight culture of *E. coli* BL21(DE3) containing a pET-22b(+) plasmid encoding *Cfr*TPL was created by inoculating 10 mL of TB_{amp} media with a single colony. This culture was shaken at 37°C and 200 rpm for ~16 hrs. Next, 10 mL of saturated culture were then used to inoculate 1 L of TB_{amp}, which was incubated at 37 °C and 200 rpm for approximately 3 hrs until optical density at 600 nm (OD_{600}) of 0.8-1.0. Cultures were removed from the incubator and cooled on ice for 30 min, followed by induction with 1 mM IPTG. The cultures were allowed to grow for additional ~16 hrs at 20 °C at 200 rpm. Cells were then harvested by centrifugation (4303 *g*, 4 °C, 15 min), and the cell pellets were stored at -20 °C overnight.

Purification. Cell pellets were thawed on ice and then resuspended in lysis buffer, comprised of enzyme storage buffer (100 mM potassium phosphate (KP_i), pH 8.0) containing 1 mg/mL *hen egg white* lysozyme (GoldBio), 0.2 mg/mL DNase (GoldBio), 1 mM $MgCl_2$, and 150 µM pyridoxal

5'-phosphate (PLP). A ratio of 4 mL lysis buffer per gram of wet cell pellet was used. Cells were resuspended and lysis was initiated by shaking for 1 hr at 37 °C. The resuspended cells were subsequently sonicated (15 min, 1 s-on/1 s-off, 30% vertical-amplitude motion). The resulting lysate was then spun at 48,300 *g* to remove cellular debris. Ni-NTA beads were then pre-equilibrated in storage buffer containing 20 mM imidazole. The resin was added to the cleared lysis supernatant and incubated under gentle agitation on ice for 1 hr. The beads were then collected in a +gravity column equipped with plastic frit, and run through a second time to collect beads. The column was washed with storage buffer (3–4 column vol) containing 20 mM imidazole. The protein was then eluted directly with 3–4 column vol of buffer containing 250 mM imidazole. The eluate containing *CfrTPL* was yellow due to the presence of the PLP cofactor. The eluate was then transferred to a centrifugal filter tube (Amicon® Ultra-15, 30 kDa molecular weight cut-off) and concentrated by centrifugation (4,000 *g*, 15 min, 4 °C). Imidazole was then removed by repeated dilution (with enzyme storage buffer) and overnight dialysis (dialysis tubing cellulose membrane, 14 kDa MW cut-off) was then performed until the imidazole concentration was less than 1 μM. Enzyme concentration was determined via the Bradford assay using bovine serum albumin as a standard. The purified enzyme was flash-frozen by dropwise pipetting into a crystallization dish filled with $N_{2(\ell)}$. The enzyme was stored in 100 mM KP_i at pH 8.0 and -80 °C. When ready to use, the frozen enzyme pellets were thawed at room temperature and centrifuged to remove potential insoluble particles (11,000 *g*, 15 min, 4 °C).

Functionality test of purified CfrTPL. Enzyme activity was estimated upon detecting quinonoid formation by treating *Cfr*TPL (100 μM) with L-Tyr (0.5 mM) and monitoring electronic absorption at λ_{max} = 505 nm under steady-state conditions (250- 600 nm, UV-2600, Shimadzu) at 25 °C. A semi-micro quartz cuvette (Starna Cells) was used (Figure S1).

Expression, purification and characterization of DsaD. *Expression*. Expression of *Streptomyces scopuliridis* DsaD, denoted here as DsaD, was carried out by procedures similar to those employed for *Cfr*TPL. Briefly, an overnight culture of *E. coli* BL21(DE3) containing a pET-28b(+) plasmid encoding DsaD was generated by inoculating 10 mL of TB_{amp} media with a single colony. The culture was shaken at 37 °C and 200 rpm for ~16 hrs. Then, 10 mL of saturated culture were used to inoculate 1 L of TB_{amp}, which was incubated at 37 °C and 200 rpm for approximately 3 hrs until OD_{600} of 0.8-1.0. Cultures were removed from the incubator and cooled on ice for 30 min, followed by induction with 1 mM IPTG. Cell growth was then continued for an additional ~16 hrs at 20 °C at 200 rpm. Finally, cells were harvested by centrifugation (4303 *g*, 15 mins, 4 °C), and the cell pellets were stored at -20 °C overnight.

Purification. Cell pellets were thawed on ice and then resuspended in lysis buffer, comprised of enzyme storage buffer (100 mM KPi buffer, pH 8.0) containing 1 mg/mL *hen egg white* lysozyme (GoldBio), 0.2 mg/mL DNase (GoldBio), 1 mM $MgCl_2$, and 150 µM PLP. A ratio of 4 mL lysis buffer per gram of wet cell pellet was used. Cells were resuspended and lysis was initiated by shaking for 1 hr at 37 °C. Resuspended cells were subsequently sonicated in a bath sonicator (15 min, 0.8 s-on/0.2 s-off). The resulting lysate was then spun at 48,300 *g* to pellet cellular debris. The resultant lysate was not further purified. In early iterations of this preparation, 10% glycerol was used throughout. However, glycerol was subsequently removed to avoid contamination of Tyr during synthesis. The lysate was then flash-frozen by dropwise pipetting into a crystallization dish filled with $N_{2(\ell)}$. The enzyme-containing lysate was stored in 100 mM KP_i at pH 8.0 and -80 °C. When ready to use, the frozen enzyme pellets were thawed at room temperature and centrifuged to remove potential insoluble particles (11,000 *g*, 15 min, 4 °C).

***Cfr*TPL activity assay.** A solution composed of ammonium chloride (500 mM), phenol (90 mM), sodium pyruvate (90 mM), KP_i buffer at pH 8.0 (100 mM), in the absence and presence of

exogenous PLP (100 μM) was generated into a 2.5 mL Eppendorf tube. The reaction was then initiated upon addition of purified *Cfr*TPL (0.1% moles of catalyst loading relative to substrate). The reaction vessel was placed in an incubator at 37 °C for 3 hrs (Figure S2A). Reaction progress was monitored by UPLC-MS (Figure S2). After reaction completion, the reaction mixture was quenched with 2 volumes of 1:1 1 M HCl: acetonitrile and centrifuged (16,162 *g*, 10 min) to remove denatured protein.

Synthesis of L-Tyr-D$_3$. L-Tyr-D$_3$ was generated according to the reaction scheme in Figure S3A. Sodium pyruvate (90 mM) was exchanged in D_2O/KPi (100 mM, pD 8.5) over 48 hrs. The H/D exchange of the $^1H^β$ methyl protons of pyruvate were monitored via 1D ^1H NMR over 48 hrs as a function of time (Figure S3B-C). The resulting fully exchanged pyruvate-D$_3$ was then added to a D_2O solution containing phenol (90 mM), ammonium chloride (500 mM, exchanged in neutral D_2O) in KP_i (100 mM, pD 8.5) in a 40 mL glass vial (Figure S3A, step 2). Step 2 was initiated upon addition of a premixed solution of H/D-exchanged PLP (final conc: 360 μM) and purified *Cfr*TPL (0.1% molar final catalyst loading relative to substrate) in D_2O. The reaction vessel was placed in an incubator at 37 °C for 2.5 hrs. Reaction progress was monitored by UPLC-MS. The reaction mixture was placed on ice for 30 min to enable product precipitation, followed by gravity filtration. The filtrate was then discarded after confirming via UPLC-MS that only a minimal amount of product was present. The insoluble product was washed with 25 mL of methanol and 25 mL of ice-cold distilled/deionized water followed by freezing and lyophilization. The final product was a white flaky powder (126 mg, 38% yield). Identity of the desired product was confirmed by 1D ^1H NMR (Figure S4).

Note that, to minimize the proton content in *Cfr*TPL reactions, enzyme, buffer, and PLP were pre-mixed, lyophilized and resuspended in D_2O. The activity of *Cfr*TPL in D_2O has been previously studied, and a modest kinetic isotope effect was detected, resulting in lower reactivity [2].

Lyophilization may further reduce enzyme function. We used the above perdeuterated materials for our reactions and, again, ^{1}H-NMR analysis confirmed complete deuteration of Tyr at C^α and C^β (Figure S4). Additional steps may be taken in the future to further optimize this protocol.

DsaD activity assay: conversion of L-Tyr-D_3 to L-Tyr-D_2. A solution composed of L-Tyr-D_3 (conc. 2.5 mM, M.W. = 184.21 g/mol), KP_i (100 mM, pH 8.0), 5% methanol, and PLP (100 μM) was generated into a 2.5 mL Eppendorf tube. The DsaD lysate (5% vol lysate/vol solution, catalyst-loading) was then added to promote the H/D exchange catalytic process. The reaction vessel was promptly placed in an incubator at 37 °C for 6 hrs and the reaction progress was monitored by UPLC-MS as a function of time (Figure S5). The process was quenched with 2 vol. of 1:1 1 M HCl:acetonitrile and the mixture was centrifuged (16,162 *g*, 10 min) to remove the precipitated DsaD.

Synthesis of L-Tyr-α-^{13}C-β,β,2,3,5,6-D_6 (QISP Tyr*).* Synthesis of QISP Tyr was performed in one chemical and two enzymatic steps, as shown in Figure S6).

Synthesis of L-Tyr-α-^{13}C-α,β,β,2,3,5,6-D_7 (L-Tyr D_7). Sodium pyruvate (90 mM) was exchanged in D_2O/KPi (100 mM, pD 8.5) over 48 hrs. The H/D exchange of the $^1H^\beta$ methyl protons of pyruvate were monitored via 1D ^{1}H NMR over 48 hrs as a function of time (Figure S3C). The resulting fully exchanged pyruvate-D_3 solution was added to a D_2O solution containing phenol-D_6 (90 mM), ammonium chloride (500 mM, pre-exchanged in D_2O) in KP_i (100 mM, pD 8.5) in a 40 mL glass vial (Figure S6, step 2). Step 2 was initiated upon addition of a mixture of PLP (360 μM) and purified *Cfr*TPL (0.1% moles of catalyst loading relative to substrate) that had been previously lyophilized and resuspended in D_2O.The reaction vessel was placed in an incubator at 37 °C for 2.5 hrs. Reaction progress was monitored by UPLC-MS. The reaction was placed on ice for 30 min to enable product precipitation, followed by gravity filtration. The filtrate was then discarded after confirming via UPLC-MS that only a minimal amount of product was present. The insoluble

product was subsequently washed with 25 mL of methanol and 25 mL of ice-cold distilled/deionized water followed by freezing and lyophilization. The final product was a white flaky powder (73 mg, 46% yield).

Isotopic exchange at $^{13}C^{\alpha}$ *for the* conversion of *L-Tyr-α-*^{13}*C-α,β,β,2,3,5,6-*D_7 *to L-Tyr-α-*^{13}*C-,β,β,2,3,5,6-*D_6 *(QISP Tyr).* A solution composed of L-Tyr-α-^{13}C-α,β,β,2,3,5,6-D_7 (conc. 2.5mM, M.W=189.62 g/mol), 5% methanol, and KP_i (100 mM, pH 8.0) was prepared in a 500 mL round-bottom flask, and the reaction was initiated upon addition of 10% v/v DsaD lysate (Figure 1C and S6, step 3). The reaction vessel was placed in an incubator at 37 °C for 6 hrs. Reaction progress was monitored by UPLC-MS. The reaction mixture was quenched with 2 vol. of acetone and centrifuged (4,300 *g*, 15 min) to remove precipitated protein. The concentrate was then loaded onto a preparative 30 g reverse-phase C18 Flash-chromatography column pre-equilibrated with water. The product was purified on an Isolera One Flash Purification system (Biotage) using a water:methanol gradient. Fractions bearing the desired product (confirmed by UPLC-MS sampling of fraction tubes) were pooled and concentrated by rotary evaporation down to 10 mL. The solution was then transferred to a pre-weighed 20 mL vial, frozen, and lyophilized. The final product (QISP Tyr) was obtained as white flaky powder (14 mg, 82% yield).

Prior to NMR characterization, QISP Tyr was further purified by FPLC followed by reverse-phase HPLC. FPLC purification was carried out via a 5 mL HiTrap Q HP column (Cytiva, flow rate 2.5 ml/min). The initial solution was water (adjusted to pH 10.1 with KOH) and QISP Tyr was eluted with a linear gradient (+1.25 %/min) of 1 M NH_4OAc (pH 10.2). The purified amino acid was then flash-frozen and lyophilized. Given the presence of some KOH in the FPLC media, the lyophilized sample contained some KOAc. Partial removal of this salt was carried out by reverse-phase HPLC (C18 BioBasic analytical column) under isocratic conditions (elution medium: methanol), followed by lyophilization. Residual amounts of acetate and methanol persisted in the final sample,

as confirmed by NMR analysis (Figure 1C). It is worth noting that NH_4OH could be used to adjust the pH of the FPLC solutions, instead of KOH, to eliminate the need for the final HPLC purification step.

General aspects of NMR data collection. All NMR data were collected on an Avance III HD 600 MHz NMR spectrometer (Bruker Biospin Corp.) equipped with a 5 mm ^{1}H [$^{19}F/^{13}C/^{15}N$] triple-resonance cryogenic probe (TCI-F) including a z-gradient. Data collection was performed at room temperature (24 ºC, uncalibrated).

1D ^{1}H and ^{13}C pulse-acquire NMR experiments for QISP Tyr characterization. Lyophilized QISP Tyr was dissolved in deionized water (600μM) containing 10% D_2O (v/v) for 1D ^{1}H and ^{13}C NMR pulse-acquire experiments in the absence and presence of ^{13}C and ^{1}H decoupling during acquisition, respectively. 1D pulse-acquire ^{1}H NMR data (Figure 1C, left) were collected in the presence of solvent suppression via the W5 excitation sculpting pulse sequence [3] with a sweep width of 10,000 Hz and 5,998 total time-domain points. A recycle delay of 5 s was employed to ensure nearly complete recovery of spin-lattice relaxation between individual scans. ^{13}C decoupling during acquisition was carried out by GARP. DSS-D_6 in deionized water with 10% D_2O (v/v) was used as external reference. 1D Pulse-acquire ^{13}C NMR data (Figure 1C, right) were collected with a sweep width of 42,613 Hz, 71,424 total point and 1.5s recycle delay. All data were processed with the MNova software (version 14.2.0), with zero-filling to 65,536 and 131,072 complex points for ^{1}H and ^{13}C spectra, respectively. An exponentially decaying window function was employed with a 3.3 and 1 Hz line-broadening for ^{1}H and ^{13}C spectra, respectively.

Preparation of GO and CAT enzyme stock solutions for LC-photo-CIDNP. Stock solutions of the GO and CAT enzymes were prepared upon dissolution of the freeze-dried powders of each individual enzyme in 10 mM KP_i at pH 7.2[4]. Concentration of the stock solutions were determined by electronic absorption spectroscopy (extinction coefficient of GO: 267,200 $M^{-1}cm^{-1}$ at 280 nm;

extinction coefficient of CAT: 912,500 $M^{-1}cm^{-1}$ at 276 nm) [5]. Enzyme stock solutions were aliquoted, flash-frozen with $N_{2(\ell)}$ and stored at -80 °C. Individual enzyme aliquots were thawed on ice cold water and the NMR sample with added GO and CAT underwent a brief incubation at room temperature prior to LC-photo-CIDNP data collection.

Preparation of Tyr-isotopolog stock solutions. Stock solutions of natural-abundance Tyr (unlabeled Tyr), Tyr-U-^{13}C-^{15}N, Tyr-α-^{13}C and QISP Tyr were prepared by dissolving the respective solid powders in distilled deionized water. Stock-solution concentrations were determined by electronic absorption spectroscopy (extinction coefficient 1,280 M^{-1} cm^{-1} at 280 nm) [6].

LC-photo-CIDNP NMR experiments under dark conditions at 14.1 T (600 MHz). High-concentration samples (10 μM) of Tyr isotopologs are required to generate the dark-conditions (LED-off) reference data necessary to determine LC-photo-CIDNP enhancement factors (Ɛ). To prepare these samples, aliquots of stock solutions of relevant Tyr isotopologs were diluted in 10 mM KP_i (pH 7.16) in the presence of 10% D_2O (v/v), to achieve a final working concentration of 10 μM. The dark (LED-off) ^{1}H-detected ^{13}C RASPRINT experiments of Figures 4D and 4E were performed at high concentration of all the amino-acid isotopologs except for natural-abundance Tyr. A long recycle delay (5 s) was used to enable nearly complete spin-lattice relaxation. Data collection included a sweep width of 10,000 Hz with 4,096 total points. Spectra were processed with MNova (version 14.1.0) and time-domain data were apodized with an exponential-decay window function (5 Hz line-broadening) followed by zero-filling to 65,536 complex points. When relevant, data acquired with identical acquisition and processing parameters were normalized upon dividing areas (or intensities) by sample concentration, to assess concentration-independent relative enhancements (see Results).

LC-photo-CIDNP NMR experiments under dark conditions at 1.88 T (80 MHz). Highly concentrated Tyr isotopologs were employed to collect reference spectra under dark conditions (LED-off). The latter were required to determine LC-photo-CIDNP enhancement factors (Ɛ). The isotopolog concentrations were: 2.15 mM Tyr-U-^{13}C, ^{15}N, 2.37 mM Tyr-α-^{13}C, and 10.75 mM QISP Tyr. All the samples used for the acquisition of dark spectra were in 10 mM KP_i (pH ~7.2). ^{1}H-detected-^{13}C RASPRINT experiments were performed on all the samples with a long 5 s recycle delay to ensure nearly complete spin-lattice relaxation. Acquisition parameters at 80 MHz with ^{13}C-RASPRINT included a 1,715 Hz sweep width and 784 total points. Spectra were processed with MNova (version 14.1.0) and time-domain data were apodized with an exponential-decay window function (5 Hz line-broadening) followed by zero-filling to 65,536 complex points. When relevant, data acquired with identical acquisition and processing parameters were normalized upon dividing areas (or intensities) by sample concentration, to assess concentration-independent relative enhancements (see Results). All dark spectra were acquired with 4,000 scans and scaled appropriately, to determine Ɛ values.

LC-photo-CIDNP NMR experiments in aqueous buffer under light conditions at 600 MHz. The LC-photo-CIDNP NMR experiments under dark (LED-off) and light (LED-on) conditions were performed on 1 μM Tyr isotopologs (Figure 2A, 2C, 4A, 4C). All NMR samples were run in the presence of either 2.5 μM fluorescein or 5 μM ATTO Thio 12; and the GO/CAT oxygen-scavenging system (2.5 mM D-glucose, 0.15 μM GO, and 0.1 μM CAT) in 10 mM KP_i, pH 7.2. The ^{1}H-detected ^{13}C RASPRINT pulse sequence was employed [7]. LC-photo-CIDNP NMR experiments under light (LED-on) conditions were carried out with the aid of a 3.5 m polymer optical fiber (POF, Prizmatix, Holon, Israel, 1.5 mm-diameter) When fluorescein was used as a photosensitizer dye, samples were irradiated with a single-chip LED (UHP-mic-LED-450, a.k.a., UHP-LED-blue; Prizmatix, Holon, Israel) equipped with a fiber adaptor [7]. The LED emission was

centered at 466 nm. When ATTO Thio 12 was used as photosensitizer, sample was irradiated with UHP-T-545-SR LED (Prizmatix, Holon, Israel) equipped with a fiber adapter. The emission was centered at 545 nm. In both cases the fiber tip was inserted into a 4 mm-diameter NMR tube, which was in turn placed into a 5 mm-diameter NMR tube carrying the sample as described [7]. Unless otherwise stated, the LED power at the optical-fiber tip was 0.58 W and 0.68 W, for the UHP-LED-blue and UHP-T-545-SR LED sources, respectively. In all experiments, 0.2 s LED irradiation time per scan was used, and 4 dummy scans were collected under dark conditions, according to known procedures [7]. LC-photo-CIDNP data on all Tyr isotopologs were collected with a sweep width of 10,000 Hz and 4,096 total points. A 0.05 s recycle delay was employed [7]. Spectra were processed with MNova (version 14.1.0), and time-domain data were apodized with an exponential-decay window function (5 Hz line-broadening) followed by zero-filling to 65,536 complex points.

LC-photo-CIDNP NMR experiments in aqueous buffer under light conditions at 80 MHz. The LC-photo-CIDNP NMR experiments under dark (LED-off) and light (LED-on) conditions were performed on 100 μM Tyr isotopologs (Figure 6). All NMR samples were run in the presence of 25 μM fluorescein and the GO/CAT oxygen-scavenging system (2.5 mM D-glucose, 0.15 μM GO, and 0.1 μM CAT) in 10 mM KP_i, pH 7.2. The ^{1}H-detected ^{13}C RASPRINT pulse sequence was employed [7]. The optical setup used at 80 MHz was the same as the one employed at 600 MHz, except that the optical fiber lengths was 2 m at 80 MHz. On the other hand, we ensured that the optical power at the fiber was 0.58 W, for the UHP-mic-LED-450 light source. In all experiments, 0.05 s recycle delay and 0.2 s LED irradiation time per scan were used, and 4 dummy scans were collected under dark conditions according to known procedures.[7] Acquisition parameters at 80 MHz with ^{13}C-RASPRINT included 1715 Hz sweep width with 784 total points. Spectra were

processed with MNova (version 14.1.0) and time-domain data were apodized with an exponential-decay window function (5 Hz line-broadening) followed by zero-filling to 65,536 complex points.

Calculation of geminate polarization of Tyr isotopologs. We took advantage of the theoretical development by Adrian[8] to predict the degree of $^{13}C^{\alpha}$ hyperpolarization in the geminate-recombination products of Tyr-U-^{13}C,^{15}N, Tyr-α-^{13}C as a function of applied magnetic field (B_0) (Figure 5). Equations and methods employed by Yang *et al.* and Li *et al.* were followed[9,10] to determine the percent geminate polarization of the Tyr isotopologs. The hyperfine coupling constants (HFCs) employed in the simulations are listed in Supplementary Table S1. All the computations were carried out via a custom-made python script (Python 3.12) at the Center for High-Throughput Computing (CHTC) of the University of Wisconsin-Madison.

$^{1}H^{\alpha}$ T_2 experiments on Tyr isotopologs. Freeze-dried powders of the appropriate Tyr isotopologs were dissolved in 10 mM KP_i (pH 7.2) to a final concentration of 1 mM (unlabeled Tyr, Tyr-α-^{13}C, QISP Tyr) and 360 μM (Tyr-U-^{13}C, ^{15}N), in the presence of 10% D_2O (v/v). A trace amount of DSS-D_6 was added as internal reference. The effect of isotope substitution on $^{1}H^{\alpha}$ resonance linewidths was quantified by assessing T_2 relaxation times. We focused on determining $^{1}H^{\alpha}$ T_2 values of Tyr-U-^{13}C-^{15}N, Tyr-α-^{13}C and QISP Tyr and unlabeled Tyr. The pulse sequence (Figure S7A) employed for this purpose, described by Yang *et al.* [9], includes solvent presaturation during the recycle delay, a perfect-echo pulse scheme [11] to eliminate $J_{^1H\text{-}^1H}$ contributions, and multiple CPMG-like cycles [12]; [13] to prolong the allowed time for ^{1}H transverse relaxation. The results of the $^{1}H^{\alpha}$ T_2 measurements are shown in Supplementary Figure S7B. The $^{1}H^{\alpha}$ T_2 of QISP Tyr is about twice as large as that of Tyr-U-^{13}C,^{15}N. Consistent with this result, the ^{1}H NMR spectrum of QISP Tyr has sharper linewidths. This result is ascribed to the multiple deuterium substitutions near the $^{1}H^{\alpha}$ site.

$^{13}C^{\alpha}$ T_1 measurements of Tyr isotopologs. A $^{13}C^{\alpha}$-RASPRINT-like pulse sequence was employed to determine $^{13}C^{\alpha}$ T_1 values as described[10]. In the case of experiments carried out at 600 MHz, the NMR samples included 250 μM of the appropriate Tyr isotopolog in 10 mM potassium phosphate buffer (pH ~7.2, H_2O) and 10% D_2O. In the case of experiments performed at 80 MHz, the samples contained 10.76 mM QISP Tyr, 5.24 mM Tyr-α-^{13}C or and 5.13 mM Tyr-U-^{13}C, ^{15}N in 10 mM potassium phosphate buffer (pH ~7.2, H_2O). The experiments at 600 MHz included a recycle delay of 10 s, an acquisition time of 0.2048 s, and 2,048 complex data points. Inversion-recovery delays of 1 ms, 50 ms, 100 ms, 250 ms, 500 ms, 800 ms, 1.5 s, 3 s, 5 s and 10 s were used, with 16 scans per delay. In the case of the experiments at 80 MHz, a recycle delay of 10 s, an acquisition time of 0.29 s, and 380 complex data points were used. Inversion recovery delays of 1 ms, 100 ms, 300 ms, 800 ms, 1 s, 1.5 s, 3 s, 5 s, 7 s and 10 s were used, with 1,024 scans per delay acquired in the case of QISP Tyr, 1,600 scans per delay acquired in the case of Tyr-α-^{13}C, and 1,600 scans per delay acquired in the case of Tyr-U-^{13}C,^{15}N. Three independent T_1 experiments were carried out at 600 MHz, and two independent experiments were carried out at 80 MHz, employing freshly prepared samples. Data were fit to

$$I(t) = I(\infty)\,(1-2e^{-\tau/T_1}) \quad , \tag{S1}$$

where I (t) is the resonance intensity at any particular inversion-recovery delay time τ, I (∞) is the intensity of the resonance at equilibrium, and T_1 is the longitudinal relaxation time. Absolute intensities were assessed with MNova 15.1.0 and normalized to the maximum intensity. Data were fit to equation S1 with Kaleidagraph 5.0.6. Resonance intensities corresponding to $\tau = 10$ s were regarded as I (∞) at both fields, assuming that at 10 s the ^{13}C magnetization has fully relaxed to equilibrium. Note that T_1 was used as the only adjustable parameter. All spectra were processed with TopSpin 4.5.0 with a 5 Hz exponential line-broadening and zero filling to 65,536 complex points. In the case of the T_1 measurements at 80 MHz, resonance intensities did not reach 1.0 even

after τ = 10 s. This behavior is likely due to slight drifts in the shimming over the course of these long experiments, given the slightly increased linewidths observed at long delay times at 80 MHz. Upon considering that Tyr is only sparingly soluble in water, we were unable to prepare highly concentrated samples, resulting in unavoidably long data collections of more than 2 days per experiment. As a result, the ^{13}C T_1 values reported at 80 MHz in Figure S8 may be slightly overestimated. Nevertheless, given that our primary aim was to compare T_1 values among various isotopologs at any given field, our conclusions are not affected by the above shortcoming.

Bacterial cell extract preparation and LC-photo-CIDNP NMR experiments on QISP Tyr in cell extracts under light conditions. A bacterial cell extract was prepared in-house from *E. coli* BL21(DE3) cells transformed with a pET11d plasmid hosting wild type drkN SH3 gene according to procedures similar to those reported by Bakke *et al.* [14], as schematically illustrated in Figure S11 (see also Fig. S11 legend). Briefly, the overnight grown bacterial cell culture was harvested by centrifugation (6,000 g, 15 min, 4 °C). Cells were resuspended on ice upon treatment with lysis buffer (50 mM Tris, 2 mM EDTA, protease inhibitors by Pierce, A32965)), pH 7.5. Resuspended cells were lysed by sonication (4 min, 1 s-on/1 s-off, 65% vertical-amplitude motion) at 4 °C with a sonic dismembrator (Fisher Scientific, model FB505, 500-Watt, 20 kHz) equipped with a probe (Ultrasonic Convertor, model CL4). The lysed cells were centrifuged (30,000 g, 15 min, 4 °C) to remove fragmented cell debris and genomic DNA. The supernatant was collected and used as cell extract. LC-photo-CIDNP NMR was performed on a 5-fold diluted cell extract (Figure S11) prepared as follows. Each 700 μl LC-photo-CIDNP NMR sample contained cell extract (140 μl), 10 mM KP_i (pH 7.2), 10% v/v D_2O, 0.15 μM GO, 0.1 μM CAT, D-glucose (2.5 mM), ATTO Thio 12 (10 μM), and QISP Tyr (10 μM). Data collection with the ^{1}H-detected ^{13}C RASPRINT pulse sequence [7] included a sweep width of 10,000 Hz with 4,096 total points and recycle delay of 0.05 s. The LED irradiation time was 0.2 s per scan. Spectra were processed with MNova (version

14.1.0), and time-domain data were apodized with an exponential-decay window function (5 Hz line-broadening) followed by zero-filling to 65,536 complex points. Linear prediction was then applied up to 65,536 data points with 2857 basis points and a coefficient of 15 via the Zhu-Bax method 23.[15]

1D ^{1}H pulse-acquire NMR experiments on QISP Tyr in bacterial cell extracts. 1D pulse-acquire ^{1}H NMR experiments (lacking any photo-CIDNP module) were performed on a 5-fold diluted bacterial cell-extract in the presence (10 μM QISP Tyr) and absence of QISP Tyr (Figure S12C). Data were collected in the presence of solvent suppression by the W5 excitation sculpting pulse sequence [3].. A sweep width of 9,678 Hz and 5,746 total points were used. The recycle delay was 1.5 s. During acquisition, ^{13}C decoupling was applied (GARP). DSS-D_6 was used as internal reference. All data were processed with the MNova software (version 14.2.0), with zero-filling to 65,536 and apodization via exponential multiplication (5 Hz line broadening). Linear prediction was applied up to 65,536 data points with 2,857 basis points and a coefficient of 15 via the Zhu-Bax method [15].

Basic features of 1D ^{1}H pulse-acquire solvent suppression (PASS) LC-photo-CIDNP pulse sequence. The 1D ^{1}H PASS-W5es LC-photo-CIDNP NMR pulse sequence used in this article is schematically illustrated in Figure 8A. The t_r and t_L parameters denote the recycle delay and LED irradiation time, respectively. The WATERGATE W5 excitation sculpting pulse sequence was used [3]. Sine-shaped 1 ms gradients (SMSQ10.100) were used, with each of them being followed by a 200 ms gradient-recovery time (τ). The following phase cycling was used: ϕ_1 = x, -x; ϕ_2 = x, x, y, y, -x, -x, -y, -y; ϕ_3= -x, -x, -y, -y, x, x, y, y; ϕ_4 = x, x, x, x, x, x, x, x, y, y, y, y, y, y, y, y, -x, -x, -x, -x, -x, -x, -x, -x, -y, -y, -y, -y, -y, -y, -y, -y; ϕ_5= -x, -x, -x, -x, -x, -x, -x, -x, -y, -y, -y, -y, -y, -y, -y, -y, x, x, x, x, x, x, x, x, y, y, y, y, y, y, y, y; ϕ_{rec} = x, -x, -x, x, x, -x, -x, x, -x, x, x, -x, -x, x, x, -x.

1D ^{1}H PASS-W5es LC-photo-CIDNP NMR experiments on Tyr and Tyr related neurotransmitters. Stock solutions of unlabeled Tyr, epinephrine hydrochloride (racemic mixture) and 3,4-hydroxy-L-phenylalanine (L-DOPA) were prepared by dissolving the respective solid powders in distilled deionized water. The concentration of each of the stock solutions was determined by electronic absorption spectroscopy at 280 nm (extinction coefficients: 1,280 $M^{-1}cm^{-1}$ for Tyr [6], 2754 $M^{-1}cm^{-1}$ for epinephrine [16] and 2,630 $M^{-1}cm^{-1}$ for L-DOPA [17]. The ^{1}H-PASS-W5es LC-photo-CIDNP pulse sequence (Figure 8A) was used for all the data in Figure 8B, C, D and Figure S14 and S15. All ^{1}H PASS-W5es LC-photo-CIDNP NMR experiments were run in the presence of the ATTO Thio 12 dye, 0.15 μM glucose oxidase (GO) and 0.1 μM catalase (CAT) enzymes, 2.5 mM D-glucose-d_{12} and 500 μM DSS in 10 mM potassium phosphate buffer (pH 7.2). The optical fiber and the experimental setup were the same as for all other LC-photo-CIDNP experiments under light conditions. A sweep width of 9,578 Hz and 4,096 total time-domain data points were used. All data were collected with a recycle delay of 0.05 s and with an LED irradiation time of 0.2 s per scan. Data were processed with the MNova software (version 15.1.0) with zero-filling to 65,536 complex points and an exponential apodization (5 Hz line-broadening). Characteristic 1D ^{1}H spectra of epinephrine hydrochloride (racemic mixture, 1 mM), L-DOPA (1 mM) and Tyr (500 μM) in 10 mM potassium phosphate buffer (pH 7.2) were acquired with the WATERGATE W5 excitation sculpting-mediated solvent suppression pulse sequence. The data are shown in Figure S15.

Determination of NMR signal-to-noise ratios and sensitivity. The signal-to-noise ratio (S/N) was evaluated as described [18], according to

$$\left(\frac{S}{N}\right) = \frac{S}{2\sigma_N} \cong \frac{2.5 \times S}{\langle N_{ptp} \rangle} \quad , \tag{S2}$$

where S, σ_N and <N_{ptp}> denote the experimentally measured signal amplitude, the r.m.s. noise amplitude and the experimentally determined peak-to-peak noise amplitude within 100 zero crossings. Note that is it customary to assume that <N_{ptp}> $\cong$ $5.0\,\sigma_N$. NMR sensitivity was estimated on a per-unit-concentration basis ($Sens_C$), according to

$$Sens_c = \frac{\left(\frac{S}{N}\right)}{[C]*\sqrt{t}} \tag{S3}$$

where [C] and t denote the concentration of the species of interest (in molarity) and the total experimental time, respectively. All $Sens_C$ values of samples undergoing LC-photo-CIDNP (light conditions) were determined at 1 μM isotopolog concentration. All other $Sens_C$ determinations were carried out at much higher concentration.

Determination of excitation rate constants of the photosensitizer dyes upon LED irradiation. The two LED sources used in this work differ in their spectral overlap with the dyes of interest, leading to different excitation efficiencies. In order to properly quantify the excitation rate constants upon LED irradiation, both LED-emission and dye-absorption profiles must be considered. We computed the average excitation rate constants ($k_{ex,avg}^{LED}$) for fluorescein and ATTO Thio 12 from the singlet ground electronic state (S_0) to the 1st singlet excited electronic state (S_1) as described[7]. We assumed uniform LED irradiation across the entire NMR tube cross-section (5 mm diameter) due to the multiple intra-NMR-tube reflections of the LED source[7], and we accounted for the extent of vertical attenuation of the LED light according to the Lambert–Beer's law. We also considered that the optical-fiber tip was positioned 0.5 cm above the NMR-detection coil region.

We used Ocean Optics High Resolution Spectrometer (Model HR-2UVV250-10) with a 1 m long and 115 μm diameter patch cord to record the emission profiles of our LEDs (UHP-mic-LED-450 and UHP-T-545-SR LED). The OceanView software was used to visualize the spectra.

The emission profile of each LED was normalized to the emission maximum and used to compute the spectral power density, $\rho(\lambda)$. The $\rho(\lambda)$ parameter is defined as the emission power per unit wavelength (across an infinitesimally small wavelength range), according to relation

$$P_{LED} = \int \rho(\lambda)\, d\lambda \quad , \qquad \text{(S4)}$$

where P_{LED} denotes the LED power at the fiber tip and $d\lambda$ denotes the wavelength interval. The P_{LED} value was set to 0.49 W for both LEDs, in the data displayed in Figure 2C. The following equation was used to compute the average excitation rate constant ($k_{ex,avg}^{LED}$)

$$k_{ex,avg}^{LED} = \int_{\lambda_1}^{\lambda_1} \int_{z_i}^{z_f} \frac{\rho(\lambda)\lambda\varepsilon(\lambda)\ln 10}{hcN_A(z_f - z_i)} \exp(-\varepsilon(\lambda)[D]zln10)dzd\lambda \quad , \qquad \text{(S5)}$$

where $k_{ex,avg}^{LED}$ denotes the excitation rate constant averaged over the NMR sample volume encompassed by the NMR receiver-coil detection region. In addition, λ_1 and λ_1 denote the shortest and longest wavelength of the dye absorption spectra overlapping with the LED emission spectra (Figure S9). Further, h denotes the Planck's constant, c is the speed of light, N_A is the Avogadro number, $z_f$and z_i are the initial and final vertical coordinates along the NMR receiver-coil detection, and $[D]$ is the concentration of the dye. A detailed derivation of equation (S5) can be found in Yang *et al* [7]. The following parameters were used to assess the excitation rate constants in Figure S9: wavelength windows λ_1–λ_2 of 420–500 nm for fluorescein; 400–660 nm and 460–660 nm for ATTO Thio-12. The z_f and z_i values were 0 mm and 22 mm, respectively. The $\varepsilon(\lambda)$ values of fluorescein and ATTO Thio 12 across the pertinent wavelength ranges were determined from Lambert–Beer's law and the respective absorption spectra. The [D] value was 2.5 μM for fluorescein and 5 μM for ATTO Thio 12, matching the experimental conditions employed to collect data on 1 μM QISP Tyr at 600 MHz. The $\rho(\lambda)$ and $k_{ex,avg}^{LED}$ values were calculated using custom-made python scripts on a MacOS computer.

Appendix:

DNA and Protein Sequences

***Cfr*TPL DNA Sequence.**

ATGAATTATCCGGCAGAACCCTTCCGTATTAAAAGCGTTGAAACTGTATCTATGATT
CCGCGTGATGAACGCCTCAAGAAAATGCAGGAAGCGGGTTACAATACTTTCCTGTTA
AATTCGAAAGATATTTATATTGACCTGCTGACAGACAGTGGCACTAACGCAATGAGC
GACAAGCAGTGGGCCGGAATGATGATGGGTGATGAAGCGTACGCGGGCAGCGAAA
ACTTCTATCATCTGGAAAGAACCGTGCAGGAACTGTTTGGCTTTAAACATATTGTTC
CGACTCACCAGGGGCGTGGCGCAGAAAACCTGTTATCGCAGCTGGCTATTAAACCT
GGGCAATATGTTGCCGGGAATATGTATTTCACTACCACCCGTTATCACCAGGAAAAA
AATGGTGCGGTGTTTGTCGATATCGTTCGTGACGAAGCGCACGATGCCGGTCTGAAT
ATTGCGTTTAAAGGTGATATCGATCTTAAAAAATTACAAAAGCTGATTGATGAAAAA
GGCGCAGAGAATATTGCGTATATCTGCCTGGCGGTGACAGTTAACCTCGCGGGCGG
CCAACCGGTCTCGATGGCTAACATGCGTGCGGTGCGTGAACTGACAGAAGCGCATG
GCATTAAAGTGTTCTACGACGCTACCCGCTGCGTAGAAAACGCCTACTTTATCAAAG
AGCAAGAGCAGGGCTTTGAGAACAAGAGCATCGCCGAGATCGTGCATGAGATGTTC
AGCTACGCCGACGGTTGTACTATGAGCGGTAAAAAAGACTGTCTGGTGAACATCGG
CGGCTTCCTGTGCATGAACGATGACGAAATGTTCTCTTCTGCCAAAGAGTTAGTCGT
GGTCTACGAAGGGATGCCATCTTATGGCGGCCTGGCAGGACGTGATATGGAAGCCA
TGGCGATTGGCCTGCGCGAAGCCATGCAGTACGAATATATTGAGCACCGCGTGAAG
CAGGTTCGCTATCTGGGCGACAAACTGAAAGCCGCTGGCGTACCGATTGTTGAACCG
GTAGGCGGTCACGCGGTATTCCTCGATGCGCGTCGCTTCTGCGAGCATCTGACGCAA
GATGAGTTCCCGGCACAAAGTCTGGCTGCCAGCATCTATGTGGAAACCGGCGTGCG
CAGTATGGAGCGCGGAATTATCTCTGCGGGCCGTAATAACGTGACCGGTGAACACC

ACAGACCGAAACTGGAAACCGTGCGTCTGACTATTCCACGTCGTGTTTATACCTACG
CACATATGGATGTTGTGGCTGACGGTATTATTAAACTTTACCAGCACAAAGAAGATA
TTCGCGGGCTGAAGTTTATTTACGAGCCGAAGCAGTTGCGTTTCTTTACTGCACGCTT
TGATTACATCTAA

Amino-acid sequence of *Cfr*TPL-6xHis construct

MNYPAEPFRIKSVETVSMIPRDERLKKMQEAGYNTFLLNSKDIYIDLLTDSGTNAMSDK
QWAGMMMGDEAYAGSENFYHLERTVQELFGFKHIVPTHQGRGAENLLSQLAIKPGQY
VAGNMYFTTTRYHQEKNGAVFVDIVRDEAHDAGLNIAFKGDIDLKKLQKLIDEKGAENI
AYICLAVTVNLAGGQPVSMANMRAVRELTEAHGIKVFYDATRCVENAYFIKEQEQGFE
NKSIAEIVHEMFSYADGCTMSGKKDCLVNIGGFLCMNDDEMFSSAKELVVVYEGMPSY
GGLAGRDMEAMAIGLREAMQYEYIEHRVKQVRYLGDKLKAAGVPIVEPVGGHAVFLD
ARRFCEHLTQDEFPAQSLAASIYVETGVRSMERGIISAGRNNVTGEHHRPKLETVRLTIP
RRVYTYAHMDVVADGIIKLYQHKEDIRGLKFIYEPKQLRFFTARFDYILEHHHHHH

***Streptomyces scopuliridis* DsaD (denoted here as DsaD) DNA Sequence**

ATGGGCAGCAGCCATCATCATCATCATCACAGCAGCGGCCTGGTGCCGCGCGGCAG
CCATATGGCTAGCATGACTGGTGGACAGCAAATGGGTCGCGGATCCGAATTCATGC
ATATTGTCACGACCCCTGTTGCGCGTCCGCTGACAGCGCAGGAGCGGACAGAGCGG
TGTGCTGCCCCGGCGTTTGGAACGGCGTTTACCGAGCACATGGTAAGTGCCAGATGG
AATCCCGAGCAAGGGTGGCATGATGCGGAATTAGTGCCCTATGGTCCGTTGTTGCTG
GACCCAGCGACAGTCGGCTTGCACTATGGGCAAGTAGTTTTCGAAGGGTTGAAAGC
ATTTCGGAGTCATACCGGAGAAGTAGCCGTGTTCCGGCCGGACGCCCACGCCGAAC
GGATGCGCGCATCCGCTCGCAGACTTATGATGCCTGAACCTCCAGAGGAATTATTTC
TTGCCGCGGTGGATGCGCTGGTGGCACAAGATCAAGAGTGGATCCCTGATGACCCT
GGTATGTCCTTATATCTGCGCCCGATTCTTTTCGCCTCAGAGCGGACGTTGGCTCTTC

GTCCAGCGCGCGAATATCGCTTTCTTTTAGTAGCATTTATAACCGAAGGATACTTTG

GACCTGCTCAAAGACCCGTTCGTGTCTGGGTGACAGATGAATACAGTAGAGCCGCA

GCGGGTGGAACGGGAGCGGCCAAGTGCGCGGGCAACTATGCTGGGAGCTTACTGGC

ACAAGAGGAGGCACAACGCAAAGGTTGTGACCAAGTGGTGTGGTTAGATCCGGTTG

AGCGTAACTGGGTGGAGGAGATGGGCGGCATGAACTTATTTTTTGTCTACGAGGCG

GGGGGGTCAGCCCGGCTGGTAACGCCGCCGCTTACTGGATCCCTGCTTCCCGGTGTT

ACCCGCGACGCCTTGTTGCGGCTTGCCCCTACGCTTGGTGTCCCAGTGTCTGAAGCT

CCGCTTTCACTTGAACAGTGGCGGGCGGACTGTGCGAGTGGCGCTATCACCGAAGTC

TTTGCCTGCGGCACTGCGGCCCGCATCTCGCCCGTGAACGAAGTTAGCACTAAGGAC

GGTTCGTGGACCATTGGGGCAGGAGCCCCGGCAGAAGGAGGGGTCGCTGCCGGTGA

AGTAACCGGTAGATTATCGGCGGCCTTATTTGGAATCCAGAGAGGCGAACTTCCTGA

TTCGCATAGTTGG ATGCGCCCTGTCTCACCTGCACGGCAATCGGCCATAACTTAA

Amino-acid sequence of DsaD-6xHis construct

MGSSHHHHHHSSGLVPRGSHMASMTGGQQMGRGSEFMHIVTTPVARPLTAQERTERC

AAPAFGTAFTEHMVSARWNPEQGWHDAELVPYGPLLLDPATVGLHYGQVVFEGLKAF

RSHTGEVAVFRPDAHAERMRASARRLMMPEPPEELFLAAVDALVAQDQEWIPDDPGM

SLYLRPILFASERTLALRPAREYRFLLVAFITEGYFGPAQRPVRVWVTDEYSRAAAGGTG

AAKCAGNYAGSLLAQEEAQRKGCDQVVWLDPVERNWVEEMGGMNLFFVYEAGGSAR

LVTPPLTGSLLPGVTRDALLRLAPTLGVPVSEAPLSLEQWRADCASGAITEVFACGTAAR

ISPVNEVSTKDGSWTIGAGAPAEGGVAAGEVTGRLSAALFGIQRGELPDSHSWMRPVSP

ARQSAIT**.**

Supplementary References

(1) Gibson, D. G.; Young, L.; Chuang, R.-Y.; Venter, J. C.; Hutchison III, C. A.; Smith, H. O. Enzymatic assembly of DNA molecules up to several hundred kilobases. *Nat. Methods* **2009**, *6* (5), 343-345.

(2) Faleev, N. G.; Demidkina, T. V.; Tsvetikova, M. A.; Phillips, R. S.; Yamskov, I. A. The mechanism of α-proton isotope exchange in amino acids catalysed by tyrosine phenol-lyase - What is the role of quinonoid intermediates? *Eur. J. Biochem.* **2004**, *271* (22), 4565-4571.

(3) Liu, M. L.; Mao, X. A.; Ye, C. H.; Huang, H.; Nicholson, J. K.; Lindon, J. C. Improved WATERGATE pulse sequences for solvent suppression in NMR spectroscopy. *J. Magn. Reson.* **1998**, *132* (1), 125-129.

(4) Lee, J. H.; Cavagnero, S. A Novel Tri-Enzyme System in Combination with Laser-Driven NMR Enables Efficient Nuclear Polarization of Biomolecules in Solution. *J. Phys. Chem. B* **2013**, *117* (20), 6069-6081.

(5) Jencks, W.; Regenstein, J. Handbook of biochemistry and molecular biology. *CRC, Cleveland, OH* **1976**, 338.

(6) Gill, S. C.; Von Hippel, P. H. Calculation of protein extinction coefficients from amino acid sequence data. *Anal. Biochem.* **1989**, *182* (2), 319-326.

(7) Yang, H.; Hofstetter, H.; Cavagnero, S. Fast-pulsing LED-enhanced NMR: A convenient and inexpensive approach to increase NMR sensitivity. *J. Chem. Phys.* **2019**, *151* (24), 245102.

(8) Adrian, F. J. Singlet-triplet splitting in diffusing radical pairs and magnitude of chemically induced electron-spin polarization. *J. Chem. Phys.* **1972**, *57* (12), 5107-5113.

(9) Yang, H.; Li, S.; Mickles, C. A.; Guzman-Luna, V.; Sugisaki, K.; Thompson, C. M.; Dang, H. H.; Cavagnero, S. Selective Isotope Labeling and LC-Photo-CIDNP Enable NMR Spectroscopy at Low-Nanomolar Concentration. *J. Am. Chem. Soc.* **2022**, *144* (26), 11608-11619.

(10) Li, S. Y.; Yang, H. M.; Hofstetter, H.; Tonelli, M.; Cavagnero, S. Magnetic-Field Dependence of LC-Photo-CIDNP in the Presence of Target Molecules Carrying a Quasi-Isolated Spin Pair. *Appl. Magn. Reson.* **2023**, *54* (1), 59-75.

(11) Aguilar, J. A.; Nilsson, M.; Bodenhausen, G.; Morris, G. A. Spin echo NMR spectra without J modulation. *Chem. Commun.* **2012**, *48* (6), 811-813.

(12) Carr, H. Y.; Purcell, E. M. Effects of diffusion on free precession in nuclear magnetic resonance experiments. *Phys. Rev.* **1954**, *94* (3), 630.

(13) Meiboom, S.; Gill, D. Modified spin-echo method for measuring nuclear relaxation times. **1958**.

(14) Bezsonova, I.; Singer, A.; Choy, W.-Y.; Tollinger, M.; Forman-Kay, J. D. Structural comparison of the unstable drkN SH3 domain and a stable mutant. *Biochemistry* **2005**, *44* (47), 15550-15560.

(15) Zhu, G.; Bax, A. Improved linear prediction for truncated signals of known phase. *Journal of Magnetic Resonance (1969)* **1990**, *90* (2), 405-410.

(16) Odajima, T.; Onishi, M. Formation of adrenochrome from epinephrine by myeloperoxidase via a free radical: Its biological significance. *Japanese Journal of Oral Biology* **1997**, *39* (4), 297-303.

(17) Yamada, S.-i.; Fujii, T.; Shioiri, T. Studies on optically active amino acids. III. Preparation of 3-(3, 4-Dihydroxyphenyl)-DL-,-D-, and-L-alanine. *Chemical and Pharmaceutical Bulletin* **1962**, *10* (8), 693-697.

(18) Ernst, R. R.; Bodenhausen, G.; Wokaun, A. *Principles of nuclear magnetic resonance in one and two dimensions*; Oxford university press, 1990.

(19) Kiryutin, A. S.; Morozova, O. B.; Kuhn, L. T.; Yurkovskaya, A. V.; Hore, P. 1H and 13C Hyperfine Coupling Constants of the Tryptophanyl Cation Radical in Aqueous Solution from

Microsecond Time-Resolved CIDNP. *The Journal of Physical Chemistry B* **2007**, *111* (38), 11221-11227.

(20) Niizuma, S.; Sato, Y.; Konishi, S.; Kokubun, H. ESR study on fluorescein semiquinone radical. *Bull. Chem. Soc. Jpn.* **1974**, *47* (9), 2121-2125.

(21) Torres, F.; Renn, A.; Riek, R. Exploration of the close chemical space of tryptophan and tyrosine reveals importance of hydrophobicity in CW-photo-CIDNP performances, Magn. Reson. 2 (1)(2021) 321–329.

(22) Jahnke, L. S.; Frenkel, A. W. Photooxidation of epinephrine sensitized by methylene blue - evidence for the involvement of singlet oxygen and superoxide. *Photochem. Photobiol.* **1978**, *28*, 517-523.

Supplementary Tables

Table S1. Hyperfine coupling constants (A) employed in the geminate-polarization calculations displayed in Figure 5.

Nucleus	A value (mT)	Ref.
**TyrO• $^{13}C^{\alpha}$**	0.6505[a]	19
**TyrO• $^{13}C^{\beta}$**	-0.492	19
**TyrO• $^{13}C^{\gamma,}$**	1.301	19
**TyrO• $^{13}C^{\delta 1}$**	-0.804	19
**TyrO• $^{13}C^{\delta 2}$**	-0.857	19
**TyrO• $^{13}C^{\varepsilon 1}$**	0.55	19
**TyrO• $^{13}C^{\varepsilon 2}$**	-0.639	19
**TyrO• $^{13}C^{\zeta 2}$**	-0.885	19
**TyrO• ^{13}CO**	-0.033	19
**TyrO• $^{1}H^{\alpha}$**	-0.026	19
**TyrO• $^{1}H^{\beta 1}$**	0.725	19
**TyrO• $^{1}H^{\beta 2}$**	0.52	19
**TyrO• $^{1}H^{\delta 1}$**	0.197	19
**TyrO• $^{1}H^{\delta 2}$**	0.209	19
**TyrO• $^{1}H^{\varepsilon 1}$**	-0.58	19
**TyrO• $^{1}H^{\varepsilon 2}$**	-0.654	19
**Fl•– ^{1}H 1,8**	0.3287	20
**Fl•– ^{1}H 2,7**	0.151	20
**Fl•– ^{1}H 4,5**	0.0889	20
**Fl•– ^{1}H 13**	0.022	20
**Fl•– ^{1}H 14**	0.019	20
**Fl•– ^{1}H 15**	0.017	20
**Fl•– ^{1}H 16**	0.009	20

[a] Scaled according to the relative hyperfine coupling constant of $^{13}C^{\alpha}$ and $^{13}C^{\gamma}$ reported in the reference and DFT-simulated hyperfine coupling constant of $^{13}C^{\gamma}$ in aqueous medium.

Supplementary Figures

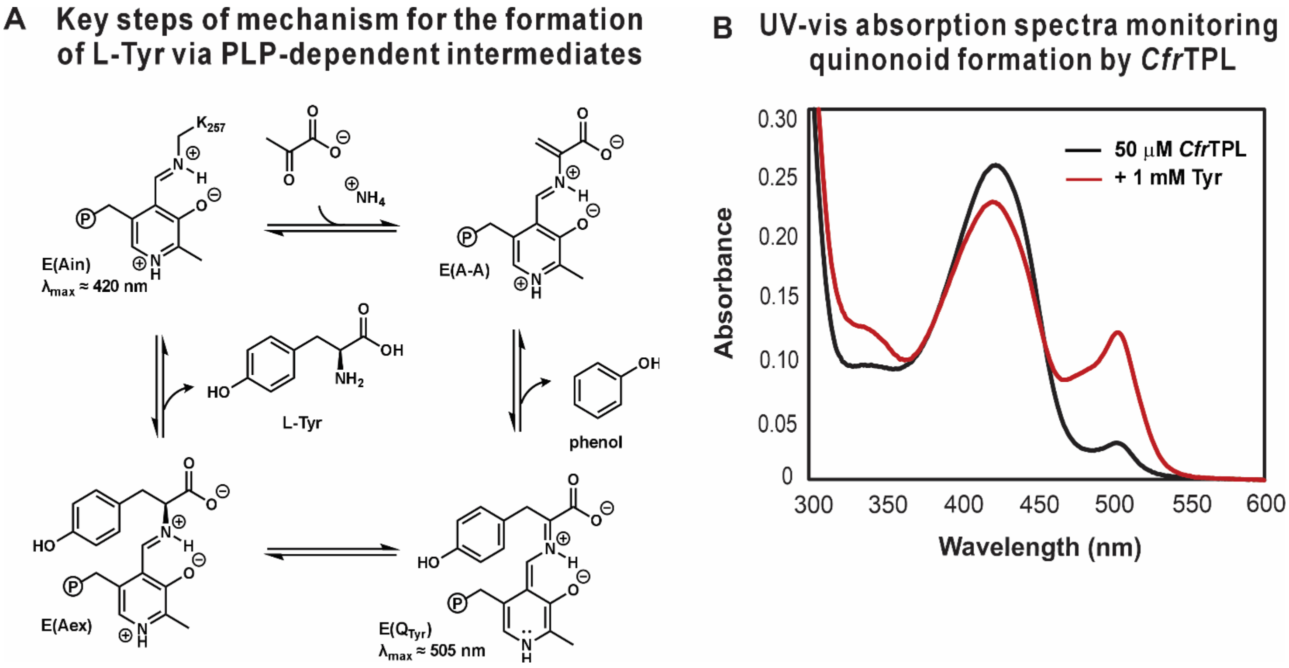

Figure S1. Electronic absorption analysis of *Cfr*TPL binding to L-Tyr for the investigation of its conformational functionality. **A.** Condensed mechanism for the formation of L-Tyr catalyzed by tyrosine phenol lyase (*Cfr*TPL). The pyridoxal phosphate cofactor is bound in the resting state as an internal aldimine, E(Ain). Binding of ammonia and pyruvate through an undetermined mechanism yields an amino acrylate, E(A-A), into which phenol adds to form a quinonoid, E(Q_{Tyr}). Tautomerization yields an external aldimine, E(Aex), which is subsequently transiminated to yield L-Tyr and return the protein to the resting state. **B.** Electronic absorption (UV/vis) spectra of *Cfr*TPL upon the addition of L-Tyr. *Cfr*TPL purifies as an internal aldimine with a small fraction of quinonoid. Addition of L-Tyr results in the formation of a strong absorption band at 505 nm. This outcome confirms that the isolated enzyme is catalytically active.

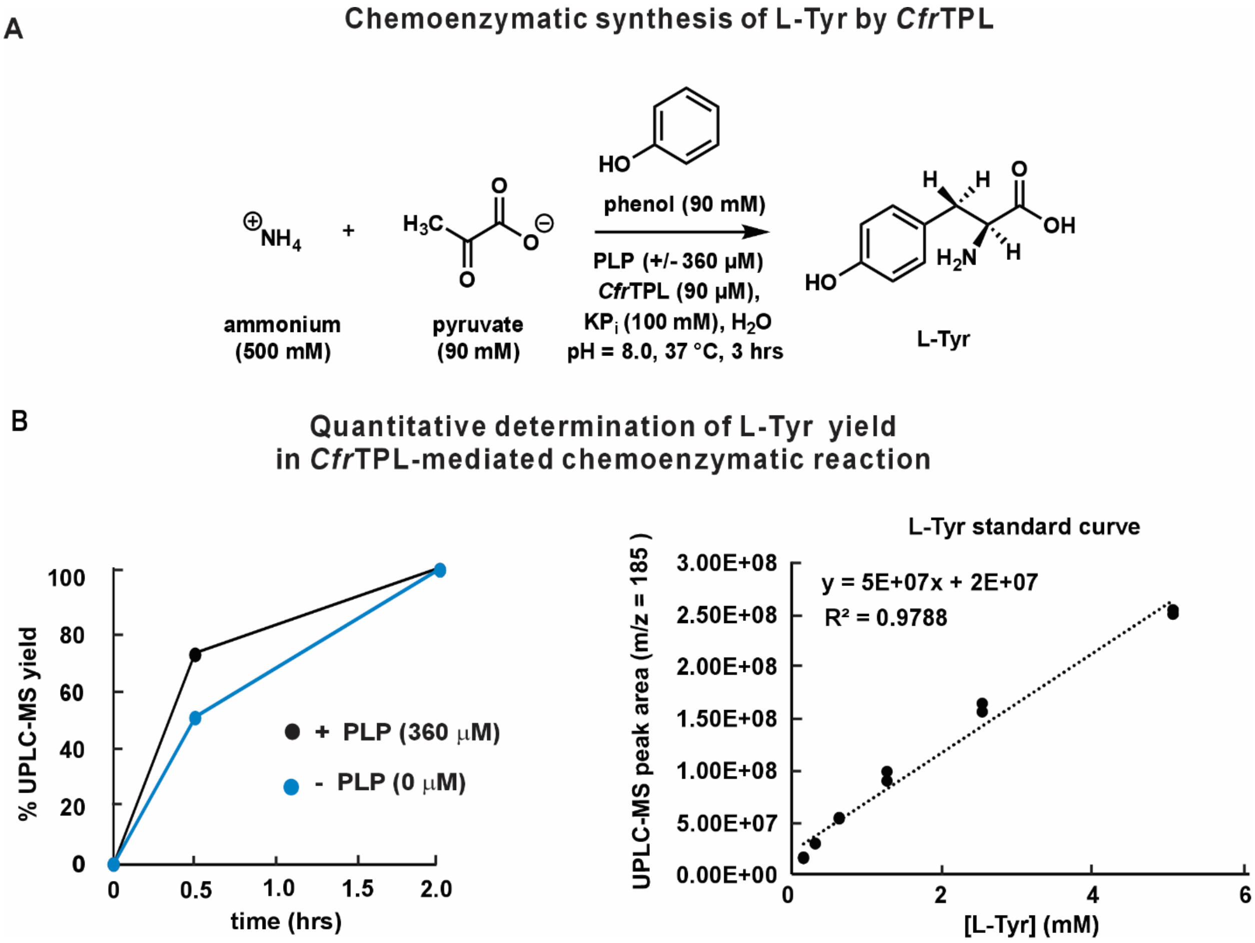

Figure S2. Optimization of *Cfr*TPL reaction conditions. **A.** Reaction scheme for screening the effects of PLP on the *Cfr*TPL-mediated synthesis of L-Tyr. **B.** Formation of L-Tyr monitored by UPLC-MS. Yield measured based on an L-Tyr standard curve (right). Addition of PLP modestly increases the rate of L-Tyr formation.

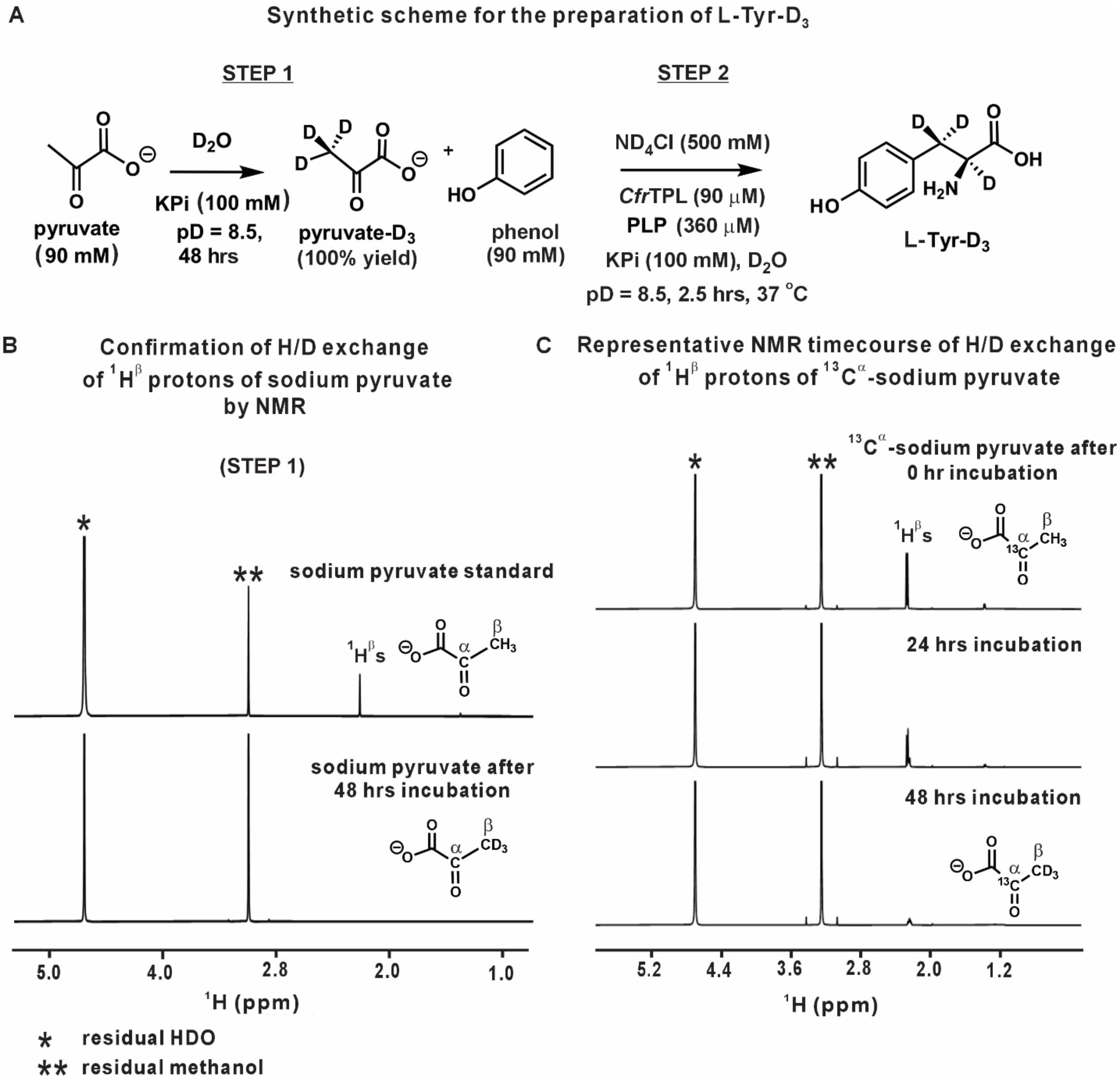

Figure S3. Preparations and H/D exchange monitoring of L-Tyr-D$_3$ A. Chemoenzymatic synthesis of L-Tyr-D$_3$ using *Cfr*TPL. **B**. Monitoring of the H/D exchange at the $^{13}C^{\alpha}$ of $^{13}C^{\alpha}$-sodium pyruvate upon incubation in D_2O buffer. The NMR intensity of the $^1H^{\beta}$ methyl resonances decrease with time over the course of 48 hrs indicating the replacement of ^1H by D.

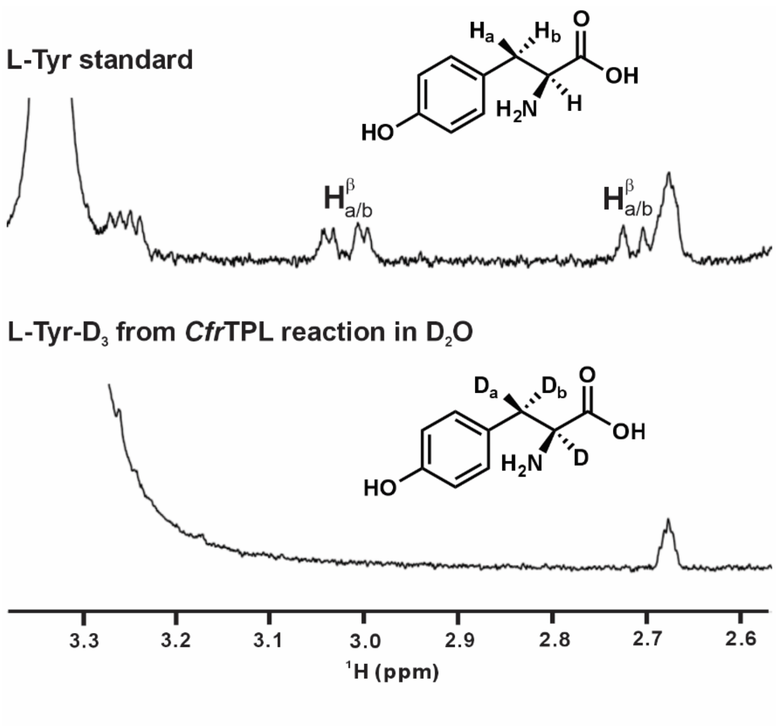

Figure S4. 1D ^{1}H NMR comparison of commercially available Tyr with L-Tyr-D$_3$ prepared in the presence of *Cfr*TPL in D$_2$O. The absence of $H^{\beta}_{a/b}$ proton resonances (bottom trace) confirms successful synthesis of L-Tyr-D$_3$.

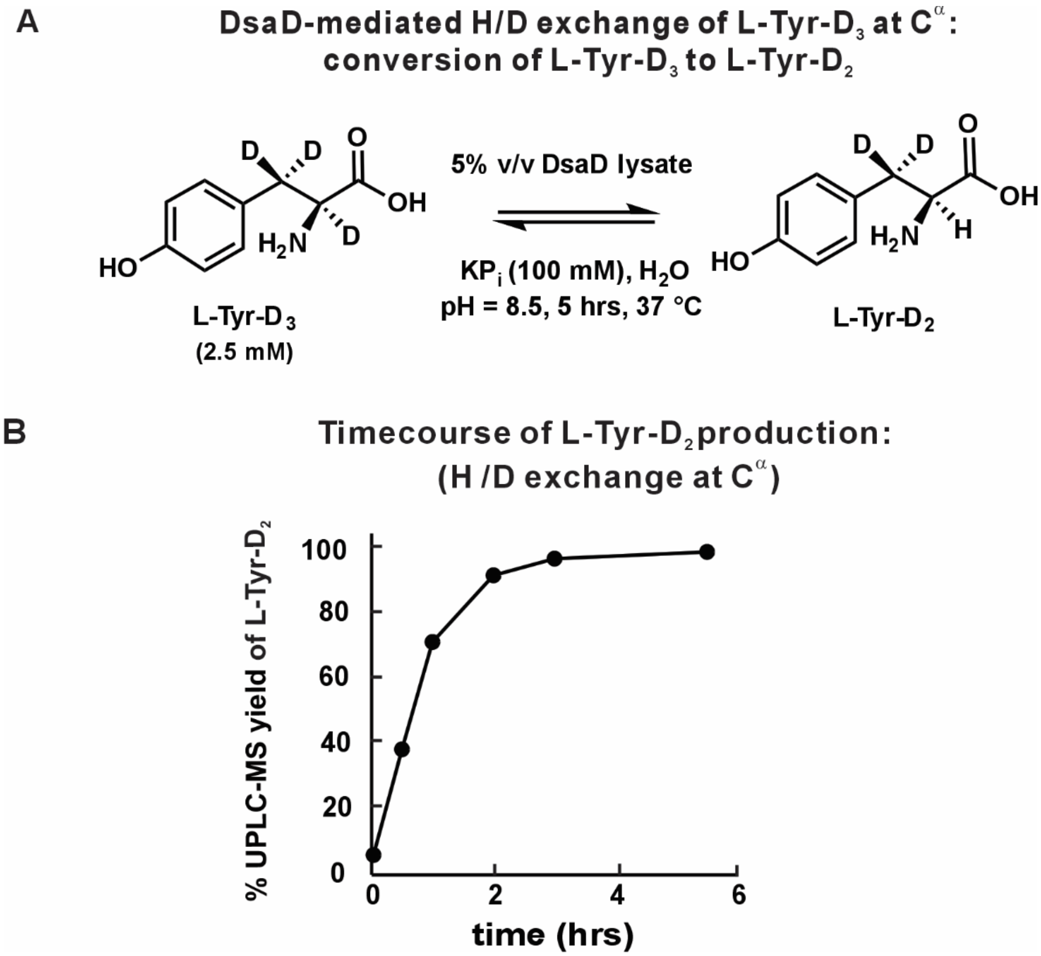

Figure S5. H/D exchange of L-Tyr-D_3 to L-Tyr-D_2 in the presence of DsaD. **A.** Chemoenzymatic reaction scheme of H/D exchange of L-Tyr-D_3 to L-Tyr-D_2. **B.** UPLC-MS analysis of DsaD mediated reaction product confirms the conversion of 95% of initial species (L-Tyr-D_3) into L-Tyr-D_2 after 5 hrs of reaction.

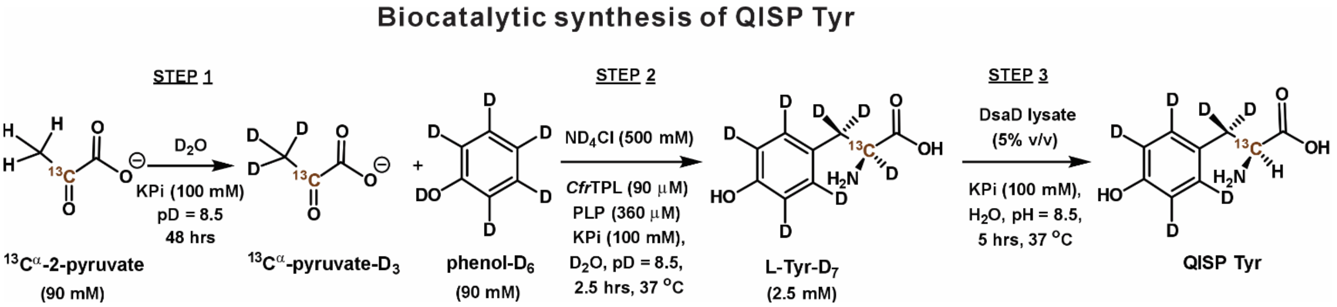

Figure S6. Chemoenzymatic synthetic scheme illustrating the production of QISP Tyr.

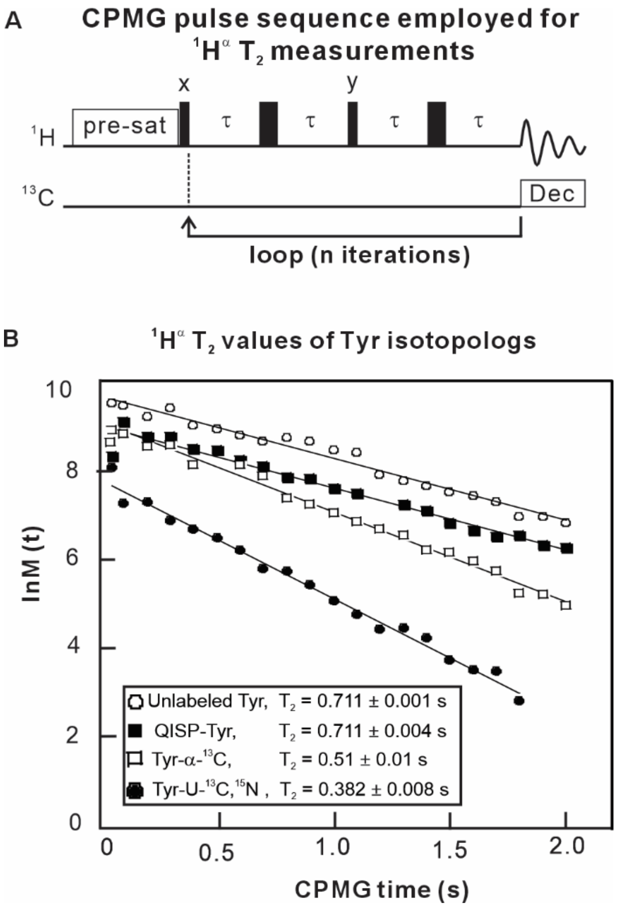

Figure S7. $^1H^\alpha$ T_2 measurements on Tyr isotopologs. **A.** NMR pulse sequence used for $^1H^\alpha$ T_2 measurements. This sequence employs a perfect-echo CPMG-like pulse scheme, and it includes solvent presaturation. The duration of each CPMG-like block was 50 ms, with the τ delay set to 12.5 ms. **B.** Plot illustrating representative $^1H^\alpha$ T_2 data. Overall T_2 values (avg ± SE for n = 2) are shown in the inset. Samples were in 10mM KP_i (pH 7.2) and 10% D_2O (see Methods). A 121.5 Hz field solvent-presaturation scheme was applied during the entire 5s recycle delay. The x-axis parameter denoted as "CPMG time" corresponds to 4τ n, where n is the number of CPMG-like iterations. Time-dependent transverse magnetization values of individual $^1H^\alpha$ resonances (M(t)) were determined from resonance areas. To determine individual T_2 values, data were fit to a single

exponential according to $\ln M(t) = \ln M(0) - R_2t$, where R_2 denotes spin-spin relaxation rate constant. All data collected on a 14.1 T (600 MHz) NMR spectrometer.

$^{13}C^{\alpha}$ T_1 relaxation time measurements of Tyr isotopologs

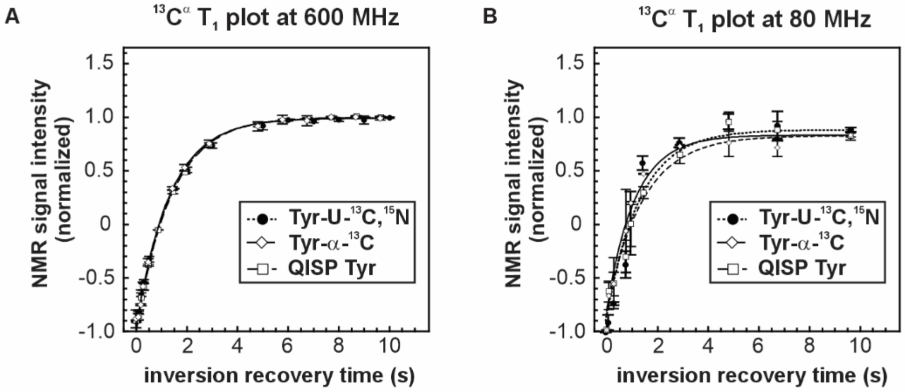

C $^{13}C^{\alpha}$ T_1 relaxation times (± standard error) at 600 MHz and 80 MHz

Applied magnetic field (B_0, MHz)	T_1 relaxation time (s)		
	Tyr-U-^{13}C, ^{15}N	Tyr-α-^{13}C	QISP Tyr
**600 (n = 3)**	1.33 ± 0.04	1.37 ± 0.03	1.44 ± 0.02
**80 (n = 2)**	1.36 ± 0.18	1.15 ± 0.01	1.48 ± 0.15

D p-value table of data in panel C at 600 MHz

Two tailed T-test at 600 MHz	p-value		
	Tyr-U-^{13}C, ^{15}N	Tyr-α-^{13}C	QISP Tyr
Tyr-U-^{13}C, ^{15}N			
Tyr-α-^{13}C	0.55		
QISP Tyr	0.14	0.21	

E p-value table of data in panel C at 80 MHz

Two tailed T-test at 80 MHz	p-value		
	Tyr-U-^{13}C, ^{15}N	Tyr-α-^{13}C	QISP Tyr
Tyr-U-^{13}C, ^{15}N			
Tyr-α-^{13}C	0.44		
QISP Tyr	0.68	0.23	

Figure S8. $^{13}C^{\alpha}$ T_1 relaxation times of Tyr isotopologs determined at 14.1 T (600 MHz) and 1.88 T (80 MHz). **(A)** plot showing the inversion-recovery data for 3 Tyr isotopologs at 600 MHz (n=3) and **(B)** plot showing the inversion recovery data for 3 Tyr isotopologs at 80 MHz (n=2). **(C)** Table showing the $^{13}C^{\alpha}$ T_1 values (± standard error) of 3 Tyr isotopologs for data collected at 600 MHz and 80 MHz. **(D)** table showing the p-values for the two-tailed Welch's T-test comparing 3 Tyr isotopologs at 600 MHz. **(E)** table showing the p-values for the two-tailed Welch's T-test comparing 3 Tyr isotopologs at 80 MHz. Note that $p \geq 0.05$ indicates that there are no statistically significant differences between the two values.

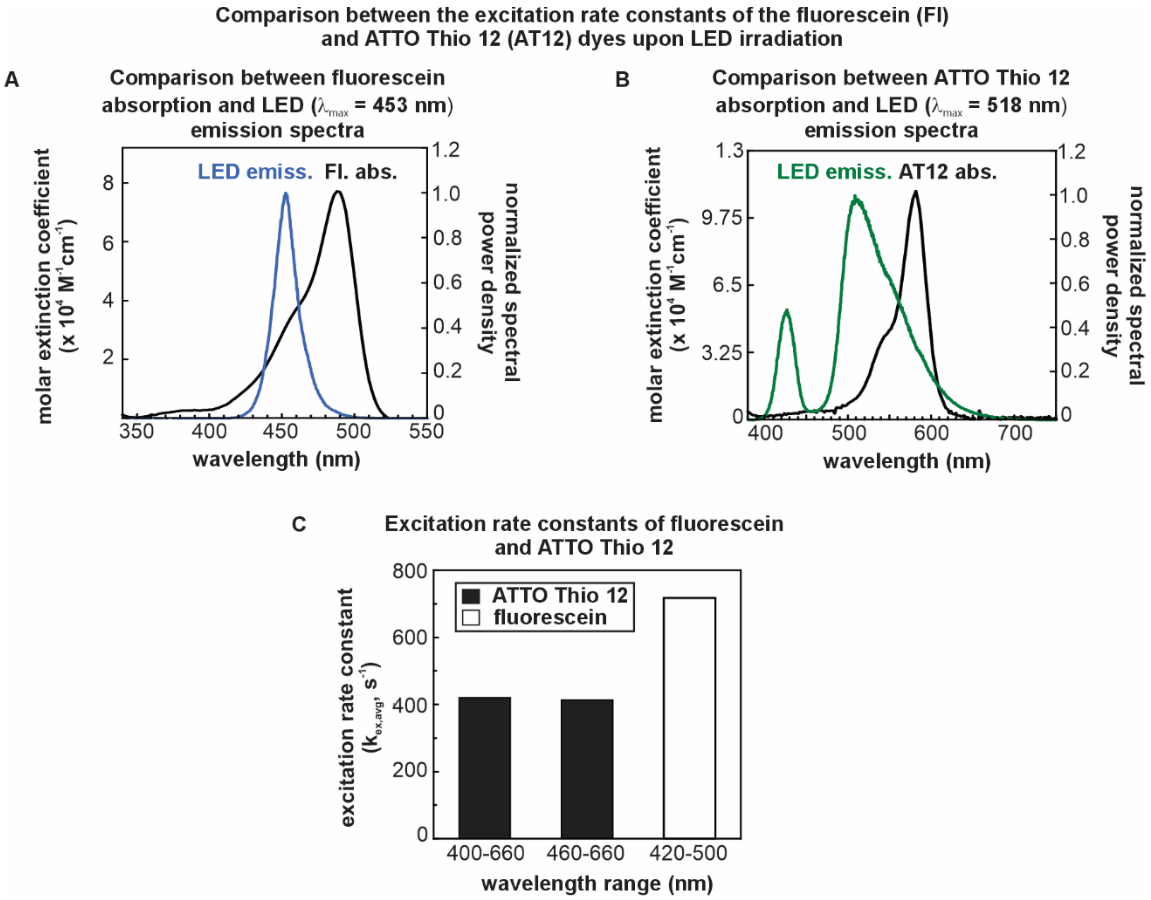

Figure S9. Spectral data and excitation rate constants of LC-photo-CIDNP photosensitizer dyes in the presence of pertinent LEDs. (A) Fluorescein absorption spectrum in water overlaid with the UHP-mic-450 LED emission spectrum. (B) ATTO Thio 12 absorption spectrum in water overlaid with the UHP-T-545-SR LED emission spectrum. (C) Computed LED excitation rate constants ($k_{ex,avg}^{LED}$ in s^{-1}) of ground-state fluorescein and ATTO Thio 12 in the presence of the respective LEDs. Two different wavelength ranges are shown for ATTO Thio 12. Although the excitation rate constant for fluorescein is larger than the one for ATTO Thio 12, with our current setup. ATTO Thio 12 may outperform fluorescein in the case of Tyr and catecholamine due to π stacking effects as described.[21]

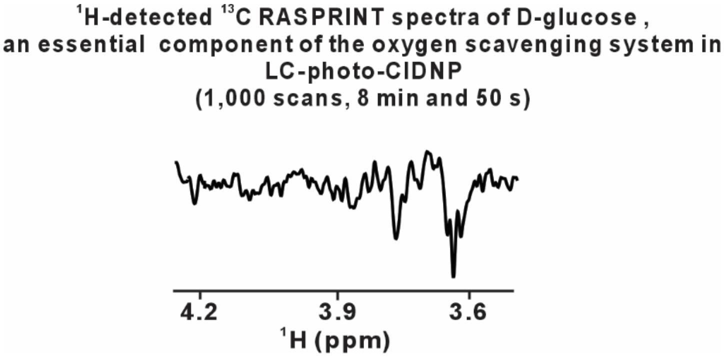

Figure S10. ^{1}H-detected ^{13}C RASPRINT spectra of 2.5mM D-glucose in 10 mM KP_i buffer (pH 7.2). The glucose resonances have been shown as negative phase to represent it similarly to the glucose resonances appeared in all LC-photo-CIDNP NMR spectra of Tyr isotopologs in buffer (n=2). Data were acquired on a 14.1 T (600 MHz) NMR spectrometer.

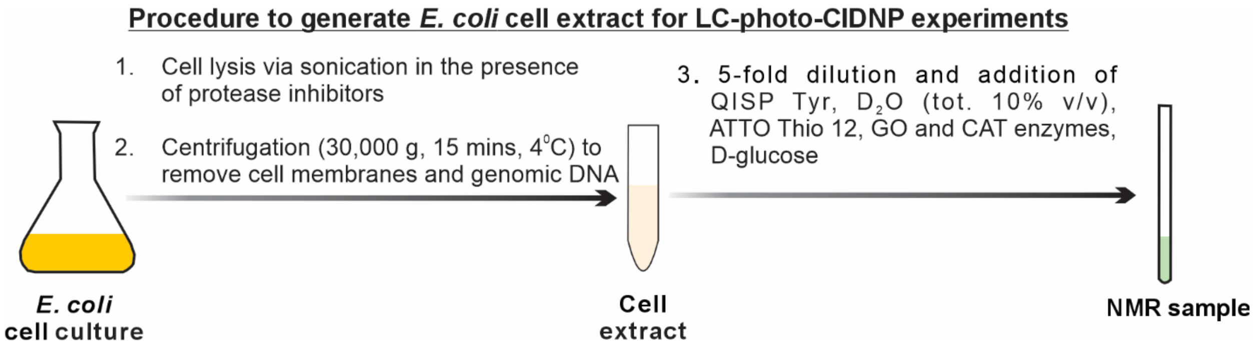

Figure S11. A schematic diagram of procedures employed to prepare *E. coli* cell extract for LC-photo-CIDNP experiments. The procedures include the lysis of overnight grown bacterial cells with the aid of probe sonicator and then centrifugation at 30,000 g for 15 min at 4 °C to isolate the cell extract from lysed cell debris like fragmented genomic content and cell wall. 10 μM QISP Tyr along with other essential components (i.e., oxygen scavenging enzyme system and ATTO Thio 12 as photosensitizer dye) were doped into 5-fold diluted bacterial cell extract to perform LC-photo-CIDNP NMR experiments.

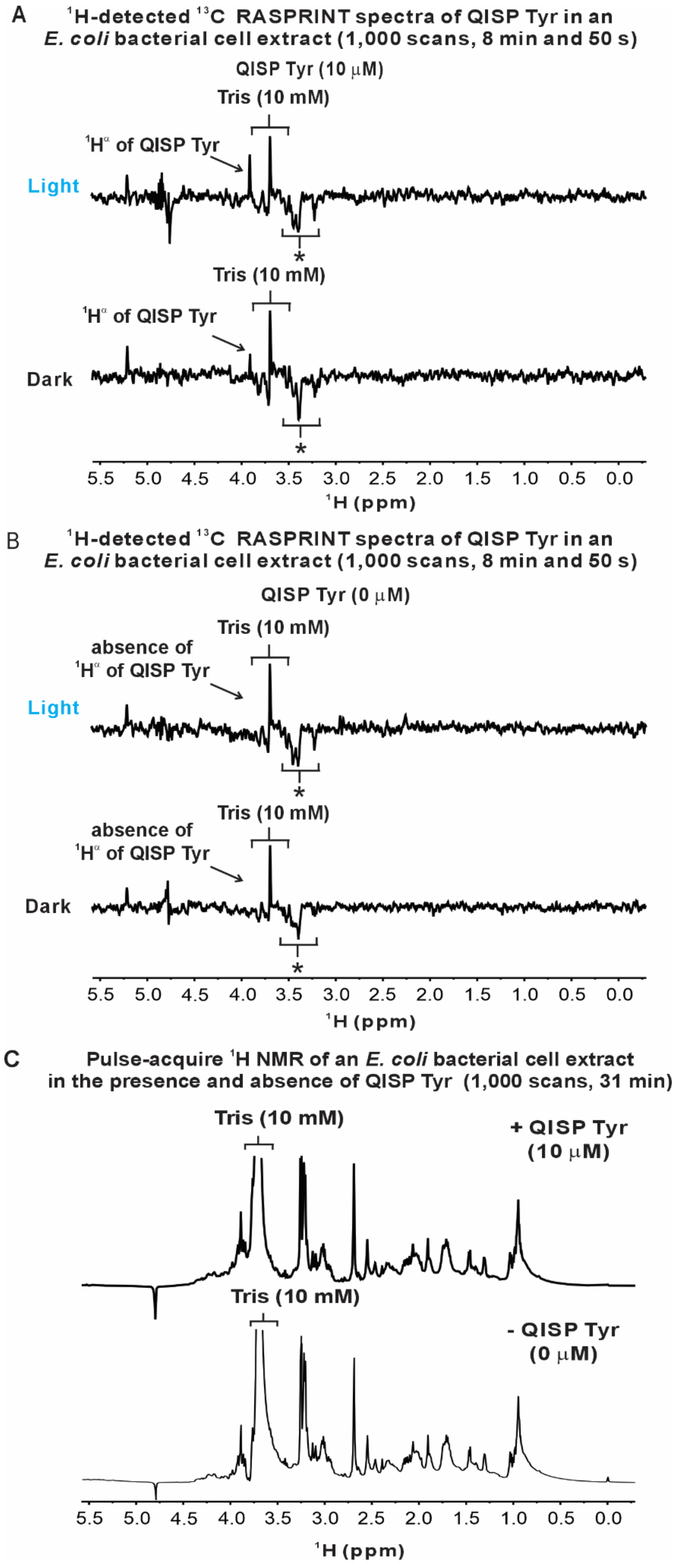
A
¹H-detected ¹³C RASPRINT spectra of QISP Tyr in an
E. coli bacterial cell extract (1,000 scans, 8 min and 50 s)
QISP Tyr (10 μM)
Tris (10 mM)
¹Hα of QISP Tyr
Light
*
Tris (10 mM)
¹Hα of QISP Tyr
Dark
*
5.5 5.0 4.5 4.0 3.5 3.0 2.5 2.0 1.5 1.0 0.5 0.0
¹H (ppm)
B
¹H-detected ¹³C RASPRINT spectra of QISP Tyr in an
E. coli bacterial cell extract (1,000 scans, 8 min and 50 s)
QISP Tyr (0 μM)
Tris (10 mM)
absence of
¹Hα of QISP Tyr
Light
*
Tris (10 mM)
absence of
¹Hα of QISP Tyr
Dark
*
5.5 5.0 4.5 4.0 3.5 3.0 2.5 2.0 1.5 1.0 0.5 0.0
¹H (ppm)
C
Pulse-acquire ¹H NMR of an E. coli bacterial cell extract
in the presence and absence of QISP Tyr (1,000 scans, 31 min)
Tris (10 mM)
+ QISP Tyr
(10 μM)
Tris (10 mM)
- QISP Tyr
(0 μM)
5.5 5.0 4.5 4.0 3.5 3.0 2.5 2.0 1.5 1.0 0.5 0.0
¹H (ppm)

Figure S12. NMR spectra of aliphatic region of 10µM QISP Tyr added to a bacterial cell extract. **A.** ^{1}H-detected ^{13}C LC-photo-CIDNP spectra of QISP Tyr (10 µM) in a bacterial cell extract under light and dark conditions (n=2). Photo-CIDNP enhances the S/N of $^{1}H^{\alpha}$ of QISP Tyr by two-fold relative to the signal present in the dark spectra. The resonance at 3.6 ppm is due to 10 mM Tris present in the bacterial cell extract as part of the lysis buffer, and has similar intensity under dark and light conditions, given that it does not undergo photo-CIDNP enhancement. **B.** ^{1}H-detected ^{13}C LC-photo-CIDNP spectra of bacterial cell extract in the absence of QISP Tyr under light and dark conditions (n=2). **C**. 1D ^{1}H NMR spectrum (pulse-acquire with solvent suppression) of 5-fold diluted bacterial cell extract in the absence and presence of 10 µM of QISP Tyr (n=2 for each experiment). The identical set of resonances in both spectra suggest that QISP Tyr is not detectable via conventional pulse-acquire 1D ^{1}H NMR. The resonances present at the aliphatic region of both spectra originate from the numerous biomolecules present in the bacterial cell extract. All data were collected on a 14.1 T (600 MHz) NMR spectrometer.

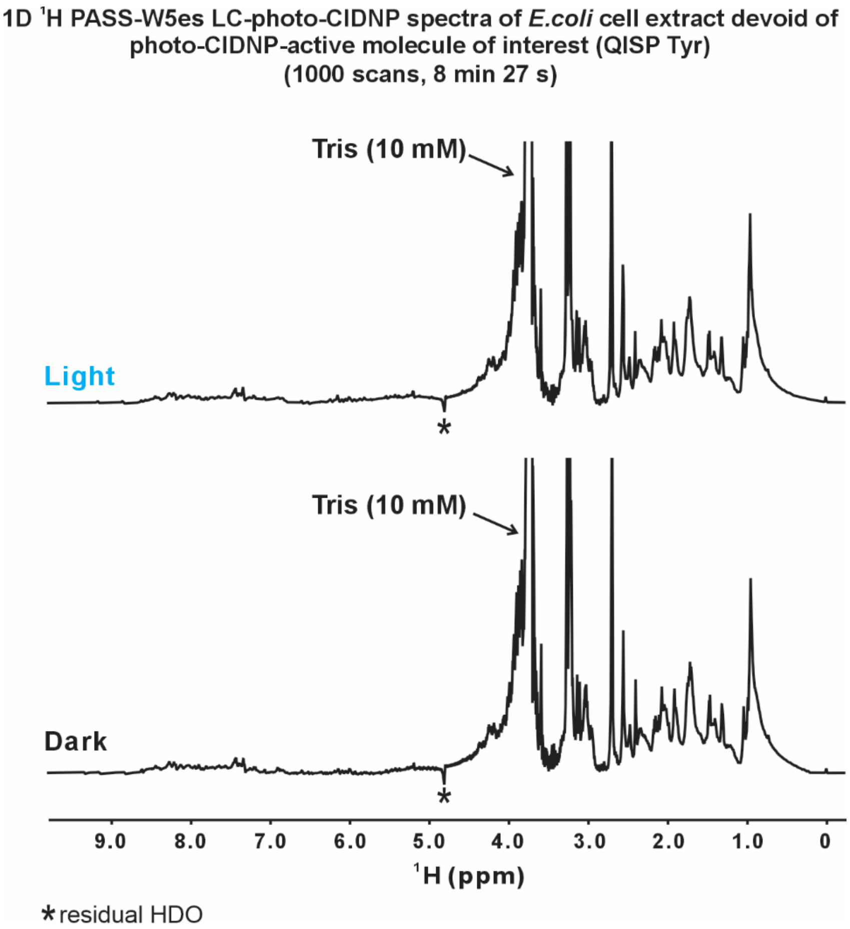

Figure S13. 1D ^{1}H PASS-W5es LC-photo-CIDNP spectra of *E. coli* cell extract in the absence of QISP Tyr. **A**. 1D ^{1}H PASS-W5es LC-photo-CIDNP spectra of 5-fold diluted *E. coli* cell extract (under light and dark conditions) in the absence of QISP Tyr (n=2). The identical set of resonances observed under both light and dark conditions show that the major endogenous components were not photodegraded nor enhanced, upon LED irradiation. The sample was dissolved in 10 mM potassium phosphate buffer at pH 7.2, and included 90% H_2O and 10% D_2O. The sample also contained 10 µM ATTO Thio 12, 0.15 µM GO and 0.1 µM CAT enzymes, and 2.5 mM D-glucose-d_{12}. The LC-photo-CIDNP experiments included a 0.05 s recycle delay and 0.2 s LED irradiation time per scan (replaced by a 0.2 s LED-off delay under dark conditions).

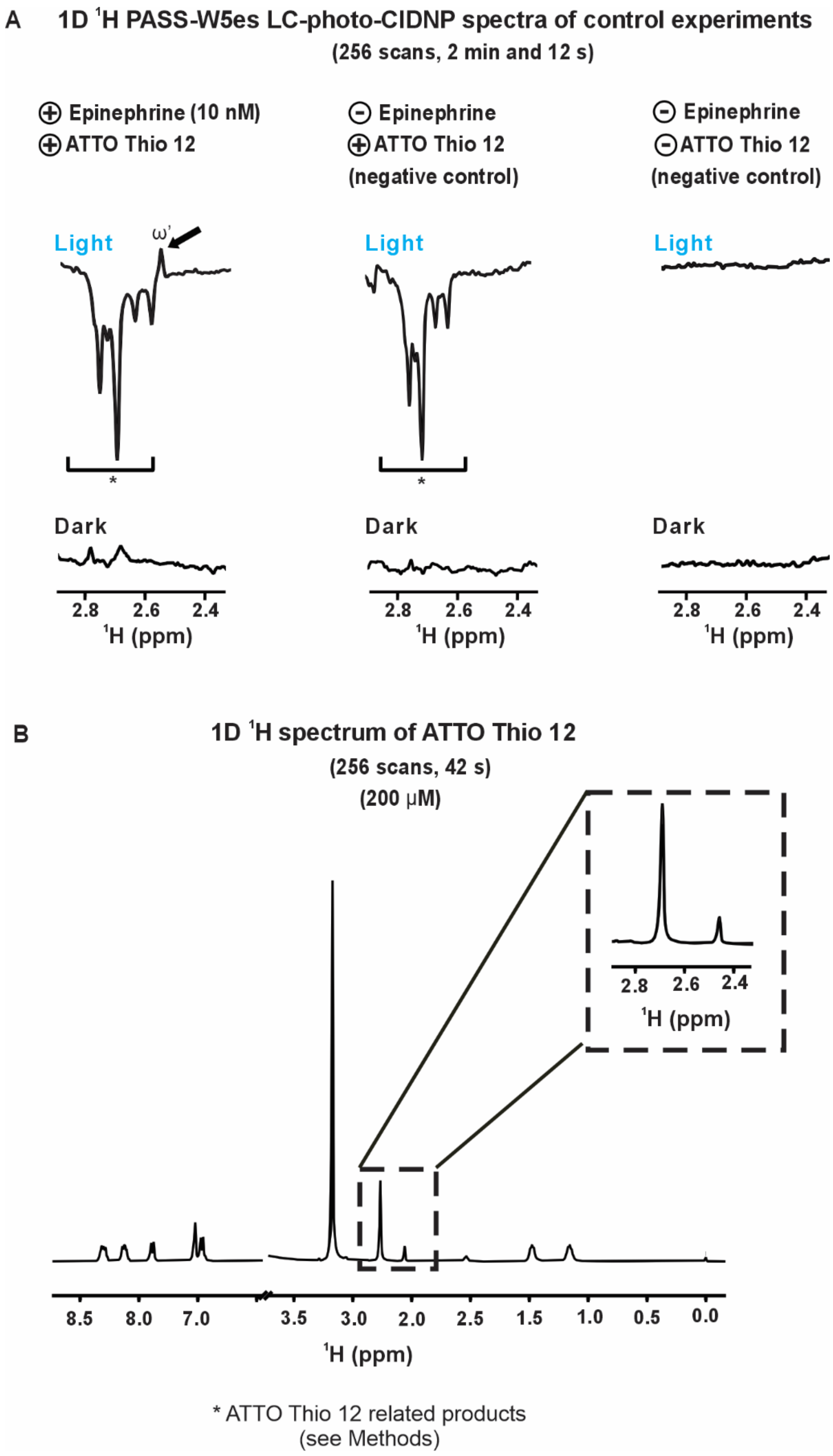

Figure S14. 1D ¹H PASS-W5es LC-photo-CIDNP control experiments confirmed that the strong emissive peaks resulted from ATTO Thio 12 degradation products. A. 1D ¹H PASS-

W5es LC-photo-CIDNP spectra (under light and dark conditions) of: (left, n=3) epinephrine (10 nM) + ATTO Thio 12 (5 μM); (middle, n=2) ATTO Thio 12 (5 μM) without epinephrine (negative control); and (right, n=2) without epinephrine and ATTO Thio 12 (negative control). All samples also contained GO (0.15 μM), CAT (0.10 μM), glucose-D_{12} (2.5 mM), phosphate buffer (pH 7.2), 500 μM DSS-D_6, and 10% D_2O. **B**. 1D ^1H NMR spectrum of 200 μM ATTO Thio 12 collected with W5 excitation sculpting and ^{13}C decoupling (during acquisition) via GARP (n=2). The recycle delays for the 1D ^1H PASS-W5es LC-photo-CIDNP and 1D ^1H NMR were 0.05 s and 1 s, respectively. Note that the zoomed-in region (2.4–2.8 ppm) shows no overlap with the emissive 1D ^1H PASS-W5es LC-photo-CIDNP resonances under light conditions, confirming that they do not arise from intact ATTO Thio 12 but, most likely, from its photo-degradation products. All the spectra shown here were acquired on a 14.1 T (600 MHz) NMR spectrometer.

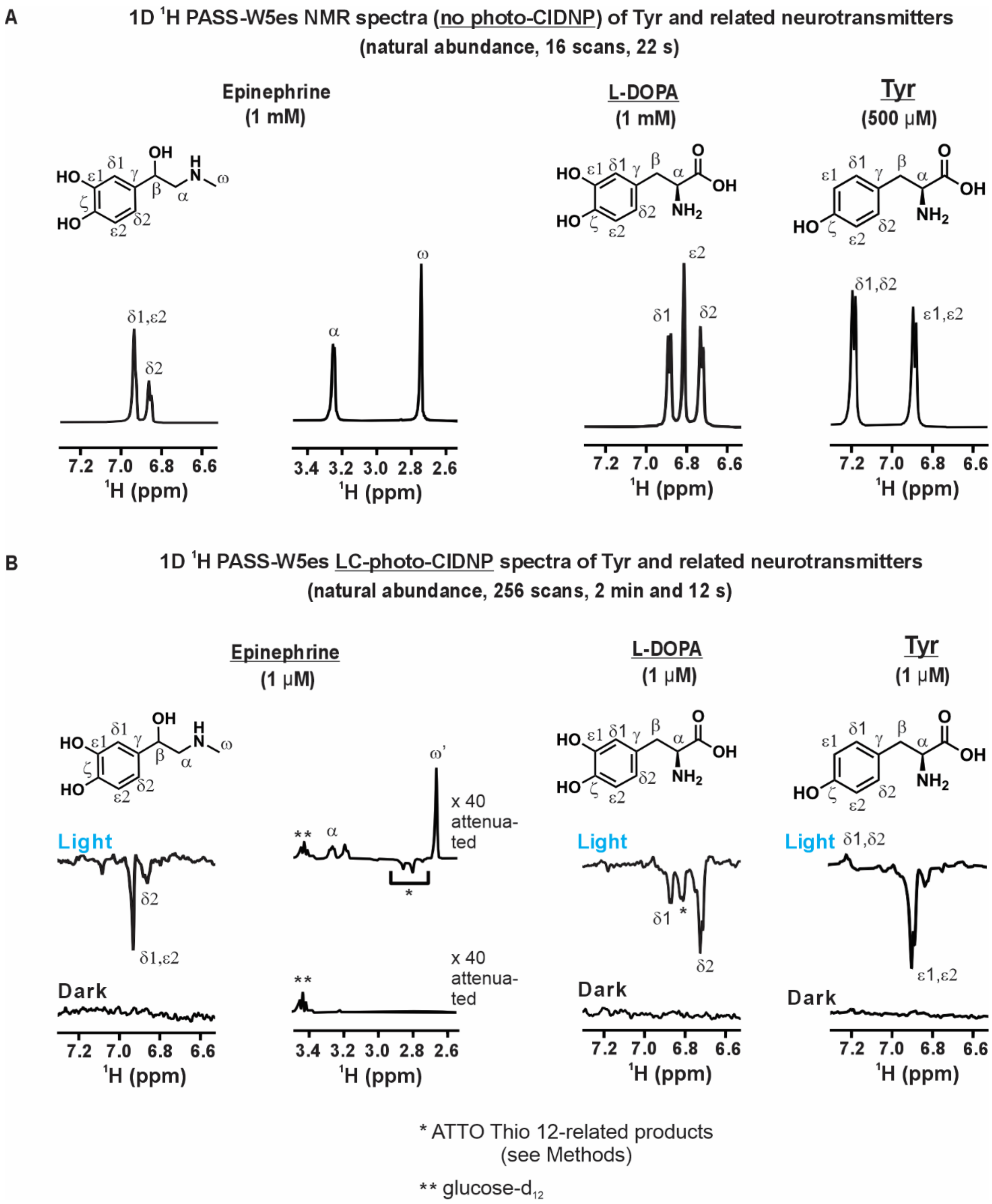

Figure S15. Comparison between conventional NMR (non-photo-CIDNP) and ^{1}H PASS-W5es LC-photo-CIDNP NMR spectra of neurotransmitters. **(A)** 1D, ^{1}H spectra of epinephrine hydrochloride (racemic mixture, 1 mM), L-DOPA (1 mM) and Tyr (500 µM), with n=2 for each species. Solvent suppression was carried out by WATERGATE W5 excitation sculpting. **(B)** ^{1}H

PASS-W5es LC-photo-CIDNP spectra of aromatic and aliphatic regions of natural-abundance epinephrine hydrochloride (racemic mixture, 1 μM); aromatic regions of L-DOPA and Tyr (1 μM), with n=2 for each species. Note the presence of (a) a ca. 0.2 ppm upfield shift of the epinephrine ω resonance (denoted as ω'), and (b) a weak new epinephrine resonance at ca. 3.2 ppm. These spectral features, which are most easily detected upon comparing epinephrine spectra in panels A and B, are likely due to photoproducts resulting from LED irradiation [22]. All experiments used ATTO Thio 12 as a dye (5 μM), a 0.05 s recycle delay and 0.2 s LED optical irradiation time per scan. All data in this figure were collected in 90% H_2O containing 10 mM potassium phosphate (pH 7.2) and 10% D_2O. All data were collected on a 14. T (600 MHz) NMR spectrometer.
